# Expanding the phylogenetic distribution of cytochrome *b*-containing methanogenic archaea sheds light on the evolution of methanogenesis

**DOI:** 10.1038/s41396-022-01281-0

**Published:** 2022-07-09

**Authors:** Ya-Fei Ou, Hong-Po Dong, Simon J. McIlroy, Sean A. Crowe, Steven J. Hallam, Ping Han, Jens Kallmeyer, Rachel L. Simister, Aurele Vuillemin, Andy O. Leu, Zhanfei Liu, Yan-Ling Zheng, Qian-Li Sun, Min Liu, Gene W. Tyson, Li-Jun Hou

**Affiliations:** 1 State Key Laboratory of Estuarine and Coastal Research, East China Normal University, Shanghai, 200241 China; 2 Centre for Microbiome Research, School of Biomedical Sciences, Queensland University of Technology (QUT), Translational Research Institute, Woolloongabba, QLD 4102 Australia; 3 Ecosystem Services, Commercialization Platforms, and Entrepreneurship (ECOSCOPE) Training Program, University of British Columbia, Vancouver, BC Canada; 4 Department of Microbiology and Immunology, University of British Columbia, Vancouver, BC Canada; 5 Key Laboratory of Geographic Information Science, Ministry of Education, East China Normal University, Shanghai, 200241 China; 6 GFZ German Research Centre for Geosciences, Section Geomicrobiology, Potsdam, Germany; 7 Marine Science Institute, The University of Texas at Austin, Port Aransas, TX 78373 USA

**Keywords:** Microbial ecology, Metagenomics

## Abstract

Methane produced by methanogenic archaea has an important influence on Earth’s changing climate. Methanogenic archaea are phylogenetically diverse and widespread in anoxic environments. These microorganisms can be divided into two subgroups based on whether or not they use *b*-type cytochromes for energy conservation. Methanogens with *b*-type cytochromes have a wider substrate range and higher growth yields than those without them. To date, methanogens with *b*-type cytochromes were found exclusively in the phylum “*Ca*. Halobacteriota” (formerly part of the phylum *Euryarchaeota*). Here, we present the discovery of metagenome-assembled genomes harboring methyl-coenzyme M reductase genes reconstructed from mesophilic anoxic sediments, together with the previously reported thermophilic “*Ca*. Methylarchaeum tengchongensis”, representing a novel archaeal order, designated the “*Ca*. Methylarchaeales”, of the phylum *Thermoproteota* (formerly the TACK superphylum). These microorganisms contain genes required for methyl-reducing methanogenesis and the Wood-Ljundahl pathway. Importantly, the genus “*Ca*. Methanotowutia” of the “*Ca*. Methylarchaeales” encode a cytochrome *b*-containing heterodisulfide reductase (HdrDE) and methanophenazine-reducing hydrogenase complex that have similar gene arrangements to those found in methanogenic *Methanosarcinales*. Our results indicate that members of the “*Ca*. Methylarchaeales” are methanogens with cytochromes and can conserve energy via membrane-bound electron transport chains. Phylogenetic and amalgamated likelihood estimation analyses indicate that methanogens with cytochrome *b*-containing electron transfer complexes likely evolved before diversification of *Thermoproteota* or “*Ca*. Halobacteriota” in the early Archean Eon. Surveys of public sequence databases suggest that members of the lineage are globally distributed in anoxic sediments and may be important players in the methane cycle.

## Introduction

Methane is an important greenhouse gas with an atmospheric concentration that has more than doubled since the start of the industrial revolution [1], which is having a profound influence on Earth’s climate. Carbon isotope studies reveal that biogenic methane production, primarily from wetlands and agricultural sources [1], is responsible for the observed rapid increase. Biological methanogenesis by methanogenic archaea (methanogens) accounts for ~74% of global methane emissions [2]. For many years it was assumed that the methanogens were phylogenetically restricted to the phylum *Euryarchaeota*, which has recently been reclassified as a superphylum consisting of three separate phyla (“*Ca*. Halobacteriota”, *Methanobacteriota* and “*Ca*. Thermoplasmatota”) in the Genome Taxonomy Database (GTDB; Release 95) [3]. Recently, metagenome-assembled genomes (MAGs) from several uncultured lineages within the *Thermoproteota* (former TACK superphylum) have been inferred to be capable of methanogenesis, greatly expanding the phylogenetic diversity of lineages possessing this metabolism. These lineages include members of the orders “*Ca*. Methanomethylicales” [4] (former phylum “*Ca*. Verstraetearchaeota”), “*Ca*. Nezhaarchaeales” [5] (former phylum “*Ca*. Nezhaarchaeota”), the classes “*Ca*. Korarchaeia” [6] (former phylum “*Ca*. Korarchaeota”) and *Nitrososphaeria* [7] (former phylum *Thaumarchaeota*). Members of the class “*Ca*. Bathyarchaeia” (former phylum “*Ca*. Bathyarchaeota”) and the order “*Ca*. Helarchaeales” (former phylum “*Ca*. Helarchaeota”) also contain methyl-coenzyme M reductase (Mcr) complex, which is the key enzyme for methane metabolism, but are suggested to more likely oxidize short-chain alkanes [8–10].

Methanogens can be divided into three groups based on substrate use: hydrogenotrophic, aceticlastic, methylotrophic, and methyl-reducing [11]. Hydrogenotrophic methanogens reduce CO_2_ to CH_4_ using electrons from H_2_ [11]. They are the most widely distributed methanogens and have been discovered in most methanogenic lineages of the *Methanobacteriota* and “*Ca*. Halobacteriota” [12, 13]. Aceticlastic methanogens generate CH_4_ and CO_2_ by disproportionation of acetate, in which the carbonyl group is oxidized to provide electrons for reduction of methyl group to methane [12]. They have been observed only in the class “*Ca*. Methanosarcinia” [14]. Methylotrophic methanogens use methylated compounds such as methylamines, methanol and methyl sulfides as carbon and energy sources. Based on studies of cultured representatives, only members of the *Methanosarcinales* are found to be capable of performing methylotrophic methanogenesis [12]. As for methyl-reducing methanogens, methyl compounds cannot be oxidized to CO_2_ but are reduced to methane using electrons derived from H_2_ or formate [12, 15]. The cultivated representatives from the *Methanomassiliicoccales*, the *Methanonatronarchaeales* and *Methanosphaera* have been shown to utilize this methyl-reducing pathway for methanogenesis [16–18]. The recently discovered “*Ca*. Methanomethylicales” and “*Ca*. Methanofastidiosa” based on metagenomic assembly are inferred to be also likely to depend on this pathway [4, 19]. Based on the difference in energy-conserving systems, all methanogenic archaea can also be classified into two main subgroups: methanogens with and without *b*-type cytochromes [12, 13]. To our knowledge, within cultivated organisms, cytochrome *b-*containing methanogens have a wider substrate range, and are able to use CO_2_ plus H_2_, acetate or methylated compounds as substrates, whereas methanogens without *b*-type cytochromes are either hydrogenotrophic or methyl-reducing [12, 13]. In addition, cytochrome *b-*containing methanogens also have higher growth yields than methanogens without *b*-type cytochromes owing to use of membrane-bound electron transport chains [12, 13]. As methanogens with *b*-type cytochromes have been exclusively found in the “*Ca*. Halobacteriota” of the *Euryarchaeota* superphylum, it has been suggested that the metabolism originated within this phylum.

Here, we present the discovery of seven MAGs containing *mcr* genes recovered from anoxic sediments that belong to novel genera within the family “*Ca*. Methylarchaceae” of the phylum *Thermoproteota*. Importantly, these putative methanogenic archaea encode cytochrome *b*-containing complexes and are predicted to conserve energy via membrane-bound electron transport chains, which expands the known phylogenetic diversity of cytochrome *b*-containing methanogens and enhances our understanding of their evolutionary history.

## Results and discussion

### Discovery of a novel archaeal lineage in wetland sediments

**Fig. 1:**
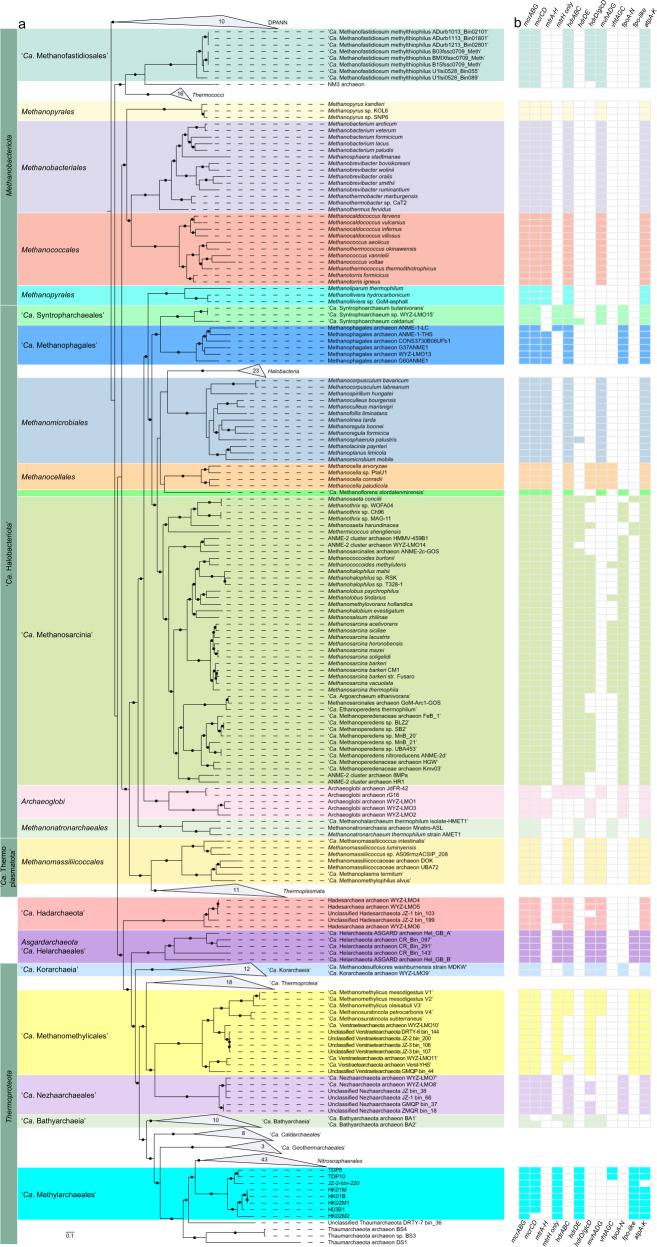
Genome tree and distribution of genes related to methane metabolism. **a** Maximum-likelihood tree of a concatenated set of 122 archaeal-specific marker genes inferred with IQTREE (LG + C60 + F + G and 1000 ultrafast bootstrapping), rooted with the DPANN superphylum, showing the placement of the “*Ca*. Methylarchaeales” (in cyan) relative to 321 archaeal genomes. Ultrafast bootstrap (BS) value ≥95 are represented by black circles. Representative *mcr*-containing archaeal lineages available in public databases are included and expanded in the tree. The lineages assigned to the *Euryarchaeota* (recently reclassified as a superphylum consisting of three separate phyla: “*Ca*. Halobacteriota”, *Methanobacteriota*, and “*Ca*. Thermoplasmatota”) are classified at the order level. **b** The phylogenetic distribution of key methane metabolism related genes. For *mtrA-H*, *fpoA-N*, *fpo-like*, and *atpA-K*, they were regarded as present if ≥80% of the subunit genes constituting these complexes were identified. For other complexes, they were regarded as present only when all subunit genes for these complexes were found.

To examine archaeal community composition and function in a mangrove ecosystem, we analyzed metagenomic data from 13 sediment samples taken from mangrove wetlands in Techeng Island of Zhanjiang and Dongzhai Harbour of Haikou, China (Supplementary Fig. 1). De novo assembly of these sequencing data (60–120 Gbp for each sample) and genome binning resulted in 242 archaeal MAGs (>70% complete; <10% contamination) (Supplementary Table 1). Five MAGs (H03B1, HK01M, HK01B, HK02M1, and HK02M2) were found to contain genes encoding a complete methyl-coenzyme M reductase complex (*mcrABCDG*) (Table 1). Based on the Genome Taxonomy Database Toolkit (GTDB-Tk) [3, 20], these MAGs were classified as a novel order within the class *Nitrososphaeria* (former phylum *Thaumarchaeota*) of the phylum *Thermoproteota* (former TACK superphylum) (Fig. 1 and Supplementary Fig. 2).

**Table 1:** Genome features of the “*Ca*. Methylarchaeales” MAGs.

Geolocation	Techeng Island	Dongzhai Harbour				Lake Towuti		Tengchong Hotspring
MAGs	H03B1	HK01M	HK01B	HK02M1	HK02M2	TDP8	TDP10	JZ-2-bin_220^a^
Proposed name	“*Ca*. Methanoinsularis halodrymi”	“*Ca*. Methanoinsularis haikouensis”			“*Ca*. Methanoporticola haikouensis”	“*Ca*. Methanotowutia igneaquae”		“*Ca*. Methylarchaeum tengchongensis”
Bin size (Mbp)	1.45	1.36	1.06	1.36	1.34	2.55	2.45	1.23
Longest contigs/scaffolds (Kbp)	75.78	57.97	39.77	74.56	76.12	29.99	282.68	75.49
Number of contigs/scaffolds	86	160	128	73	91	570	141	54
N50 value	25,415	11,242	12,847	26,542	19,004	5614	53,503	30,160
Number of predicted genes^b^	1607	1490	1151	1437	1446	3291	2850	1354
Completeness (%)^c^	96.60	95.63	90.78	92.72	94.42	94.69	99.51	97.09
Contamination (%)^c^	0.97	1.46	0.97	2.43	2.91	0.97	0.97	0.97
Strain heterogeneity (%)^c^	0.00	0.00	0.00	33.33	25.00	100.00	0.00	0.00
GC (%)	42.51	40.68	41.11	40.81	36.64	38.78	38.95	39.00

^a^Hua et al. [7].

^b^Inferred with Prodigal.

^c^Based on lineage-specific marker sets determined with CheckM.

Comparative analyses revealed that the McrA sequences from these MAGs are distantly related to extant sequences in the NCBI nr database (≤74.2% amino acid identity (AAI)), but have 78.0–80.0% AAI to that of “*Ca*. M. tengchongensis” (IMG-M database accession no. Ga0263250) [7]. These *mcrA* genes were also found to be homologous to genes (>85.4% AAI) detected in two metagenomes in IMG database generated from sediments of Lake Towuti, Indonesia (Supplementary Fig. 1 and Supplementary Table 2). Two additional related MAGs (TDP8 and TDP10, Table 1) encoding the complete Mcr complex were subsequently recovered from these metagenomes. For these MAGs (with exception of HK01M), the *mcrABG* operon and other genes related to methane metabolism were located on long contigs (≥11,476 bp) whose sequence composition features were consistent with their corresponding genomes (Supplementary Fig. 3), supporting the accurate assignment of these contigs to each MAG. The estimated genome size range for the seven MAGs recovered was 1.06–2.55 Mbp with total number of coding sequences ranging from 1151 to 3291. We examined vertical distribution of these MAGs in sediment cores of two sampling sites and found that their relative abundance increased gradually as depth increased from 15 to 100 cm (Supplementary text; Supplementary Fig. 4). Subsequent searches of public sequencing databases using the 16S rRNA and *mcrA* gene sequences annotated in these MAGs identified related species in freshwater lake sediments, hot springs, mangrove wetlands, rice paddy soils, hydrothermal vents, and deep-sea sediments distributed in different regions of the world (Supplementary text; Supplementary Table 3 and Supplementary Fig. 5).

Phylogenomic analysis using 122 concatenated archaeal-specific marker proteins revealed that the seven MAGs and “*Ca*. M. tengchongensis” formed a distinct lineage that is sister to the order *Nitrososphaerales* (Fig. 1a and Supplementary Fig. 2b). Phylogenetic analyses of the 16S and 23S rRNA genes recovered from these MAGs supported the novelty of this lineage (Supplementary Table 4 and Supplementary Fig. 2a), with pairwise nucleotide comparisons of 16S rRNA genes revealing an identity of 79.1–87.3% to publicly available *Nitrososphaeria* genomes (Supplementary Table 5). The seven MAGs belonging to the novel lineage had an AAI of 44.0–52.3% to all other genomes of the *Nitrososphaeria* (Supplementary Table 6), further supporting their classification as a separate order [21, 22]. Collectively, these phylogenetic analyses indicate that these MAGs represent four different genera of the recently described family “*Ca*. Methylarchaceae” within a novel order—designated here as “*Ca*. Methylarchaeales” (Fig. 1a and Supplementary Fig. 2 and Supplementary Tables 5 and 6). H03B1, HK01M, HK01B, and HK02M1 represents one genus (69.7–80% AAI to other MAGs), HK02M2 represents the second (68.9–80% AAI to other MAGs), TDP8 and TDP10 represent the third (70.2–82.5% AAI), and “*Ca*. M. tengchongensis” represents the fourth (68.9–82.5% AAI); the former three genera are named here “*Ca*. Methanoinsularis”, “*Ca*. Methanoporticola”, and “*Ca*. Methanotowutia”, respectively.

### The “*Ca*. Methylarchaeales” are potentially methyl-reducing methanogens with *b-*type cytochromes

**Fig. 2:**
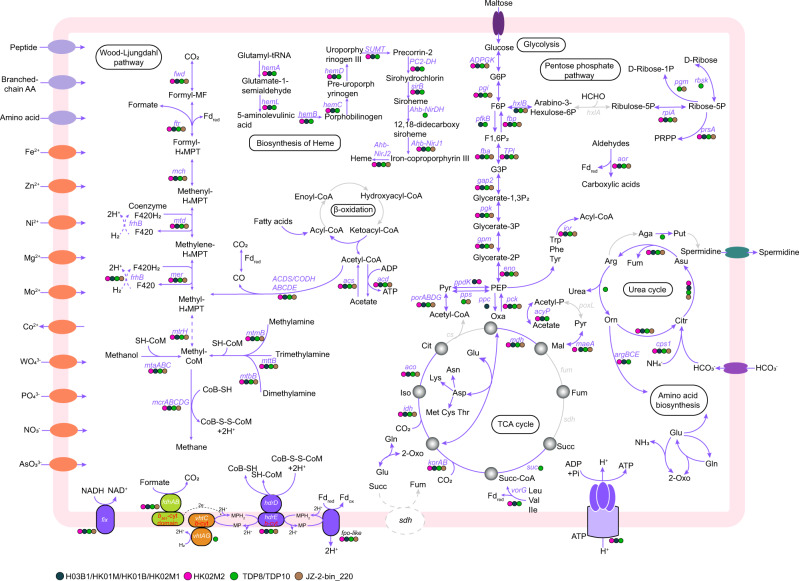
Proposed metabolic pathways in the “*Ca*. Methylarchaeales”. Genes found in H03B1/HK01M/HK01B/ HK02M1 (blue-green dots), HK02M2 (pink dots), TDP8/TDP10 (green dots), and JZ-2-bin_220 (brown dots) or missing from all bins (gray) are indicated. Genes associated with these pathways and their full name are provided in Supplementary Table 7. MP Methanophenazine, Fd ferredoxin, *b-cyt b*-type cytochrome.

Annotation of the eight “*Ca*. Methylarchaeales” MAGs confirmed genes involved in archaeal methane metabolism (Supplementary Table 7 and Fig. 2), including those encoding the Mcr complex (*mcrABG* and auxiliary genes *mcrCD*), and the ATP-binding protein AtwA (component A2) required for Mcr activation [23]. The “*Ca*. Methylarchaeales” harbor genes for methane production from methanol and methylamines (*mtaA*, *mtbA*, *mttB*, *mtbB*, and *mtmB*) (Supplementary Table 7 and Fig. 2), suggesting that the “*Ca*. Methylarchaeales” have potential to perform methyl-reducing methanogenesis, as previously suggested for “*Ca*. M. tengchongensis” [7], and members of the orders *Methanomassiliicoccales* [15], “*Ca*. Methanofastidiosales” [19] and “*Ca*. Methanomethylicales” [4]. All of the “*Ca*. Methylarchaeales” MAGs encoded a tetrahydromethanopterin (H_4_MPT) S-methyltransferase subunit H (MtrH), and either a MtrX or MtrA, that are homologous to those of *Methanosarcina barkeri* (Supplementary Table 7). Phylogenetic analysis revealed that the “*Ca*. Methylarchaeales” MtrH subunits are more closely related to a MtrH (BP07_RS03240) of *Methermicoccus shengliensis* than to the MtrH subunits of *Methanosarcina* (Supplementary Fig. 6). It is likely that the “*Ca*. Methylarchaeales” MtrH may be involved in methyl transfer directly to H_4_MPT, as previously shown in *M. shengliensis* for utilization of methoxylated aromatic compounds [24]. The absence of a complete gene operon for Mtr complex suggests that the “*Ca*. Methylarchaeales” cannot use the CO_2_ reduction or aceticlastic pathway for methanogenesis.

In contrast to the “*Ca*. Methanomethylicales”, all genes for the Wood-Ljundahl pathway (WLP) and acetyl-CoA decarbonylase/synthase: CO dehydrogenases (ACDS/CODH) are also present in all the genomes (Supplementary Table 7 and Fig. 2). However, we did not identify the energy-converting hydrogenase complex and F_420_-reducing hydrogenase complex, both of which are required for the oxidation of the methyl groups to CO_2_ via the WLP [12]. This suggests that the “*Ca*. Methylarchaeales” cannot utilize the methylotrophic pathway for methanogenesis. Similar to methyl-reducing methanogens of the *Methanonatronarchaeales* [17], function of the defective WLP remains a mystery.

**Fig. 3:**
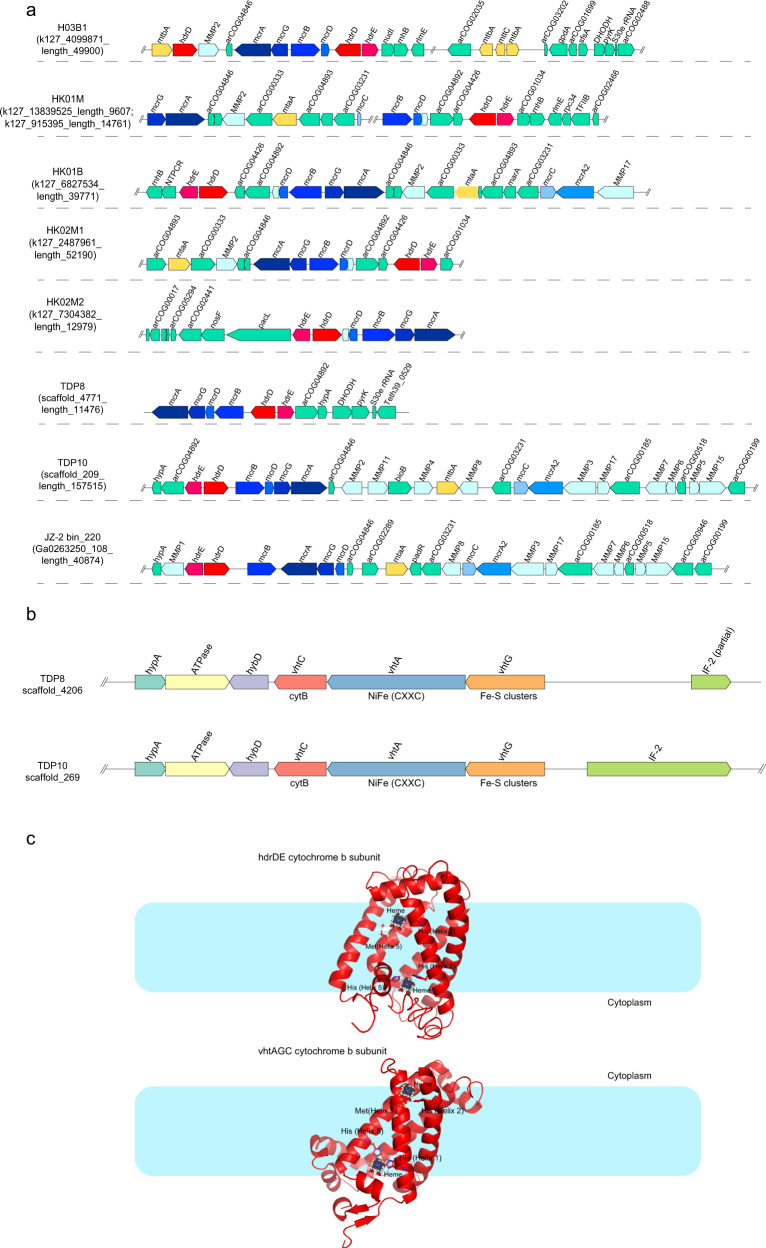
Gene composition and structural model of HdrDE and VhtAGC complexes in the “*Ca*. Methylarchaeales”. **a** Gene composition of contigs/scaffolds containing the gene cluster of heterodisulfide reductase (HdrDE) complex. Genes related to methane metabolism are highlighted with red, blue, yellow, and cyan. The hdrDE complex gene cluster is always adjacent to mcrABDG operon. **b** Gene composition of methanophenazine-reducing hydrogenase (VhtAGC) complex. Genes for VhtAGC were collocated on the same contig/scaffold, forming a transcriptional unit. **c** Structural model of *b*-type cytochromes in HdrDE and VhtAGC complexes showing the proposed heme ligation.

The “*Ca*. Methylarchaeales” MAGs contain one or two copies of a gene encoding heterodisulfide reductase subunit D (HdrD) (Supplementary Fig. 7 and Supplementary Table 7), one of which was co-located with a *b*-type cytochrome gene (Fig. 3a and Supplementary Fig. 7), which is similar to the *hdrDE* operon of *Methanosarcina barkeri* [25]. The *b*-type cytochromes in the HdrDE-like complex of the “*Ca*. Methylarchaeales” are integral membrane proteins with five transmembrane helical segments that harbor a nitrate reductase gamma subunit domain (PF02665) (Fig. 3c and Supplementary Figs. 7 and 8). Sequence analysis of these *b*-type cytochromes revealed two histidine residues located in Helix 2 of these proteins in all the “*Ca*. Methylarchaeales” genomes, two histidine residues located in Helix 5 for H03B1, and single histidine and methionine residues located in Helix 5 for “*Ca*. Methanotowutia” and “*Ca*. Methanoinsularis” (Supplementary Fig. 7b and Fig. 3c). These residues are suggested to be involved in the binding of two heme groups [26], similar to the NarI of *E. coli* [27] and HdrE of *M. barkeri* [25]. It is assumed that the two heme groups ligated to histidine or methionine residues of Helix 1 and Helix 5 are on the periplasmic and cytoplasmic side of the membrane bilayer respectively, and are responsible for electron transfer. In addition, the *hdrDE* operon is adjacent to the *mcrABDG* operon in all the “*Ca*. Methylarchaeales” MAGS (Fig. 3a), supporting their role in methanogenesis for these microorganisms. Collectively, these findings strongly indicate that members of the “*Ca*. Methylarchaeales” are *b*-type cytochrome-containing methanogens that use the HdrDE complex to reduce the heterodisulfide CoM-S-S-CoB of Coenzymes M and B generated in the final step of methanogenesis [28] (Fig. 2).

We identified a homolog of a 11-subunit NADH-quinone oxidoreductase complex in each “*Ca*. Methylarchaeales” genome (Supplementary Table 7) whose gene cluster resembles to the F_420_H_2_ dehydrogenase (Fpo) found in *Methanosarcina* [29] (Supplementary Fig. 9b). Phylogenetic analysis of the large subunit revealed that the “*Ca*. Methylarchaeales” complex is more closely related to the Fpo and Fpo-like complexes of *Methanosarcinales* and *Methanomassiliicoccales* than to group 4 [NiFe] hydrogenases (Supplementary Fig. 10). The absence of the typical [NiFe]-binding motifs in the catalytic subunit excludes the possibility that the complex is a group 4 [NiFe] hydrogenase (Supplementary Fig. 9a). In addition, the complex also lack the FpoF subunit required for binding and oxidation of F_420_H_2_ [15]. This suggests that this Fpo-like complex is unable to interact with F_420_H_2,_ and instead may use reduced ferredoxin as an electron donor, similar to its proposed role for the *Methanomassiliicoccales* [15] and *Methanosaeta thermophila* [30]. In six MAGs from “*Ca*. Methanoinsularis”, “*Ca*. Methanoporticola”, and “*Ca*. M. tengchongensis”, genes for soluble methyl viologen-reducing hydrogenase/heterodisulfide reductase complex (MvhADG/HdrABC) and methanophenazine-reducing hydrogenase complex (VhtAGC) are missing. It is extremely unlikely that genes encoding all MvhADG/HdrABC and VhtAGC complex subunits are present in these near-complete genomes but were missed by sequencing. Thus, it is proposed that these microorganisms may use the Fpo-like complex directly to accept electrons from reduced ferredoxin, and subsequently channel these electrons to the HdrDE complex coupled to the reduction of CoM-S-S-CoB (Fig. 2), as shown previously for *Methanosaeta thermophila* [30]. The reduced ferredoxin may be produced by some unidentified hydrogenases or an unknown pathway. The H03B1 MAG also encodes a formate dehydrogenase subunit A gene *(fdhA)* co-located with a *fdhB* gene (Supplementary Table 7) and a putative *b*-type cytochrome with five transmembrane helices and a prokaryotic *b561* domain (PF01292) binding two heme groups (Supplementary Fig. 11c) that is similar to FdhC of *E. coli*. “*Ca*. M. tengchongensis” contained *fdhAB* genes, with the *fdhB* gene collocated with a gene for a cytochrome *b561* with four transmembrane helices and two heme groups (Supplementary Fig. 11b). It is likely that these microorganisms may be able to use formate dehydrogenase to reduce methanophenazine pool which could then transfer electrons to the membrane-bound HdrDE complex (Fig. 2). We identified a geranylfarnesyl diphosphate synthase homolog in each “*Ca*. Methylarchaeales” genome. Phylogenetic analysis revealed that these enzymes cluster together with the geranylfarnesyl diphosphate synthase of *M. mazei*, likely suggesting that the “*Ca*. Methylarchaeales” may be able to synthesize methanophenazine, as previously shown in *M. mazei* [31] (Supplementary Fig. 12).

The “*Ca*. Methanotowutia” (TDP8 and TDP10) MAGs encode the small and large subunits for a [NiFe] active site-containing hydrogenase co-located with a gene for membrane-spanning *b561* domain (PF01292) cytochrome *b* (Fig. 3b), which is similar to the operon of VhtAGC complex found in *Methanosarcina* with cytochromes [12]. The *b*-type cytochrome harbors five transmembrane helices, with histidine or methionine residues located in Helix 1, 2, 5 for the ligation of two heme groups (Supplementary Fig. 11a). It has been proposed that the VhtA is guided to the cell membrane with the help of twin-arginine signal peptide of VhtG and its [NiFe] active site faces periplasmic side [32, 33]. As a result, two H^+^ ions generated by H_2_ oxidation are released into the periplasm while two electrons are transferred to heme groups of VhtC through Fe-S clusters of VhtG [12, 34]. Furthermore, the electron carrier methanophenazine connects VhtAGC with HdrDE, and its reduction and reoxidation results in the release of two additional H^+^ ions into the periplasm (Fig. 2) [34, 35]. Altogether, four electrogenic protons are generated in the system, which can be used to drive the synthesis of one ATP via an archaeal A-type ATP synthase. The HdrDE complex that receives electrons from the methanophenazine can be used to reduce CoM-S-S-CoB (Fig. 2), enabling the coupling of methane production with energy conservation. This is the first report of a VhtAGC complex and an HdrDE complex found in an *mcr*-containing archaeal lineage outside the *Euryarchaeota* superphylum (Fig. 1) and indicates that “*Ca*. Methanotowutia” may be capable of performing H_2_-dependent methyl-reducing methanogenesis. The membrane-bound electron transport chain is more efficient than electron bifurcation that is used by methanogens without cytochromes [12].

**Fig. 4:**
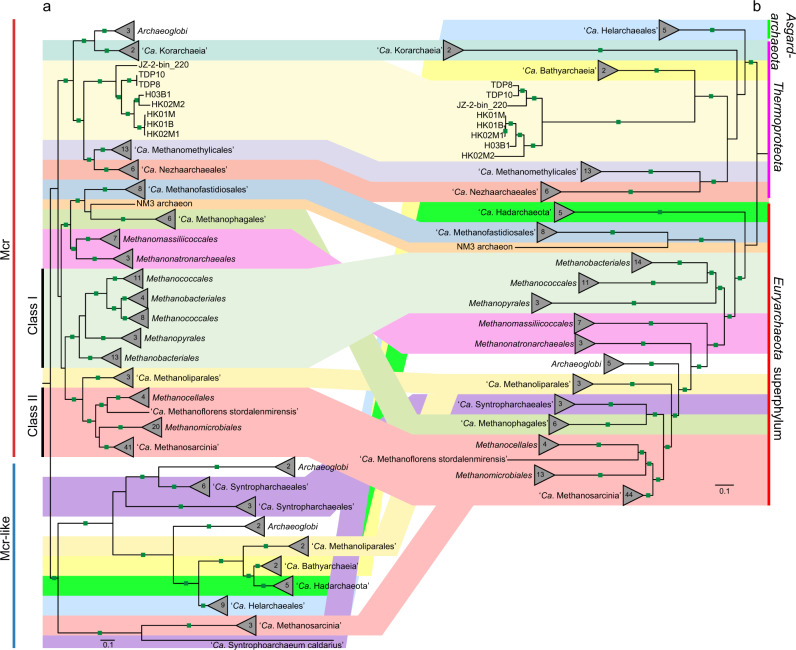
Phylogeny of the Mcr/Mcr-like complex showing the relationship with their species tree. **a** Maximum-likelihood tree (IQ-TREE, LG + C60 + F + G) based on an alignment of concatenated McrABG/McrABG-like subunits from 167 archaeal genomes. The Mcr-like complex is found in short-chain alkane-oxidizing archaea. **b** Maximum-likelihood tree (IQTREE, LG + C60 + F + G) based on concatenated 122 archaeal-specific marker proteins using the same genomes with those of Mcr/Mcr-like tree. Ultrafast bootstraps values ≥95 are indicated with green filled squares.

Sequence analysis revealed that key conserved residues of the McrA sequences of the “*Ca*. Methylarchaeales”, including the binding sites for F_430_ cofactors, coenzyme M, and coenzyme B [36], are the same as those in McrA sequences of members of the *Euryarchaeota* superphylum, with exception that the cysteine at site α452 is replaced with an alanine or serine (Supplementary Fig. 13 and Supplementary Table 8). Phylogenetic trees of concatenated and individual McrABG were reconstructed, showing that the “*Ca*. Methylarchaeales” encode canonical Mcr complexes that cluster with those of putative methane-metabolizing archaea and are divergent from those of short-chain alkane-oxidizing archaea (Fig. 4 and Supplementary Fig. 14). These results support the view that the “*Ca*. Methylarchaeales” metabolize methane.

We also explored the possibility that the “*Ca*. Methylarchaeales” may be able to oxidize methane. In reported anaerobic methanotrophic archaea (ANME), methane oxidation is coupled to the reduction of several electron acceptors (nitrate, sulfate or metal oxides). Known ANME are predicted to utilize canonical terminal respiratory reductases or multi-heme c-type cytochromes (MHCs) to transfer electrons to a syntrophic partner microorganism [37], metal oxides [38, 39] or humics [40]. We could not identify any terminal reductases or MHCs in the “*Ca*. Methylarchaeales” genomes. Previous studies have hypothesized that formate or acetate might act as potential syntrophic electron carriers between methane-oxidizing archaea and their partners [41, 42], and members of the “*Ca*. Methylarchaeales” possesses the genetic potential for the production of these electron carriers. However, to our knowledge, these electron-transferring mechanisms have never been experimentally verified for ANME. Collectively, these analyses suggest that these “*Ca*. Methylarchaeales” are more likely methanogens, although empirical studies are required to confirm this.

Similar to all described methanogens [15], the “*Ca*. Methylarchaeales” do not encode a complete tricarboxylic acid cycle, with citrate synthase, fumarase and succinate dehydrogenase absent from these MAGs. The “*Ca*. Methylarchaeales” lack a canonical pyruvate kinase for glycolysis (Supplementary Fig. 15 and Supplementary Table 7). However, pyruvate-water dikinase or pyruvate phosphate dikinase in gluconeogenesis may replace pyruvate kinase to catalyze the reversible interconversion of phosphoenolpyruvate and pyruvate, as shown in cultivated methanogens *Methanomassiliicoccales* [15]. The identification of sugar transport proteins and a variety of extracellular and intracellular carbohydrate-active enzymes (CAZymes) including glycoside hydrolases (EC 3.2.1.1 and 5.4.99.16) and glycosyltransferases (EC 2.4.1, 2.4.1.83, and 2.4.99.18, etc.) in the “*Ca*. Methylarchaeales” (Supplementary Fig. 15) suggests that they may be able to utilize sugars as an alternative carbon and energy source, as previously hypothesized for the “*Ca*. Methanomethylicales” and “*Ca*. Bathyarchaeia” [4, 8]. However, comparative genomics revealed that cultured methanogens that do not utilize sugars also encode similar proteins (Supplementary Fig. 15) [12, 13], and they may instead be involved in biosynthetic pathways. In addition, peptide and amino acid transporters, and enzymes related to peptide fermentation including extracellular peptidases, endopeptidases, 2-oxoglutarate ferredoxin oxidoreductase (*kor*), 2-ketoisovalerate ferredoxin oxidoreductase (*vor*), indolepyruvate ferredoxin oxidoreductase (*ior*), and pyruvate ferredoxin oxidoreductase (*por*) are present in both the “*Ca*. Methylarchaeales” and cultured methanogens (Supplementary Fig. 15). Nevertheless, to our best knowledge, peptide fermentation has never been reported in these isolated methanogens to date. Thus, the genes may be involved in assimilation and metabolism of amino acids in the “*Ca*. Methylarchaeales” and other newly discovered uncultured methanogens [4, 8, 12].

### Evolution of the *b-*type cytochrome-containing methanogens

The rapid increase in the number and diversity of MAGs has greatly expanded the known diversity and distribution of Mcr genes in archaea. To investigate the evolutionary history of the Mcr complexes in methanogens, we inferred the phylogeny of concatenated McrABG subunits based on all *mcr*-containing archaeal genomes available in public databases. In accordance with previous studies [43, 44], lineages in Class I and Class II methanogens within the *Euryarchaeota* superphylum appear congruent between McrABG and species trees while H_2_-dependent methylotrophic methanogens *Methanomassiliicoccales* and *Methanonatronarchaeia*, and methanotroph “*Ca*. Methanophagales” (ANME-1) are not (Fig. 4). The results were further supported by the phylogeny of the six conserved markers (m4–m9) in this (Supplementary Fig. 16) and previous studies [44]. These markers are solely present in archaea containing Mcr or Mcr-like complexes and suggested to be involved in activation, folding and assembly of Mcr subunits [44]. The Mcr genes of “*Ca*. Methanomethylicales” and “*Ca*. Korarchaeia” within the phylum *Thermoproteota* were previously suggested to be acquired via HGTs, since they are closely related with those of methylotrophic methanogens of the *Euryarchaeota* superphylum in McrABG tree [44]. However, analyses including our “*Ca*. Methylarchaeales” MAGs and several others with an Mcr complex revealed good congruence between the concatenated McrABG, m4-m9 genes, and the genome-based trees for the lineages within the *Thermoproteota* (including the “*Ca*. Methanomethylicales”, “*Ca*. Korarchaeia”, “*Ca*. Nezhaarchaeales”, and our “*Ca*. Methylarchaeales”; Fig. 4 and Supplementary Fig. 16) support vertical inheritance and evolution independent of the *Euryarchaeota* superphylum. Wide distribution of *mcr* genes in archaea (Supplementary Fig. 17 and Supplementary Table 9) and their congruence with the genome-based tree for many lineages within the *Euryarchaeota* superphylum and the *Thermoproteota* suggest that these genes likely have originated before the divergence of these two major archaeal lineages.

Recently, amalgamated likelihood estimation (ALE) has been used to estimate presence probability of McrA in each internal node in a rooted archaeal species tree, supporting the presence of McrA with high confidence in the common ancestor of Class I and Class II methanogens, “*Ca*. Methanofastidiosales”/“*Ca*. Nuwarchaeales” in *Euryarchaeota* superphylum, as well as “*Ca*. Methanomethylicales”, “*Ca*. Korarchaeia”, and “*Ca*. Nezhaarchaeales” in the Thermoproteota [45]. Compared to the previous study [45], our ALE results support the presence of McrA with high confidence [presence probability (pp) >0.9] at the basal node of “*Ca*. Methanomethylicales”, “*Ca*. Nezhaarchaeales”, “*Ca*. Korarchaeia”, and “*Ca*. Methylarchaeales” in the *Thermoproteota* (Supplementary Fig. 17), suggesting an earlier origin of Mcr complex in *Thermoproteota*. The difference is likely attributed to the addition of “*Ca*. Methylarchaeales”. Confidence in evolutionary inferences from ALE analyses will require expansion of genome coverage of some of the poorly represented or yet-to-be-discovered Mcr-containing lineages. A previous study showed that an ancestral McrA sequence were more closely related to McrA from “*Ca*. Methanodesulfokores washburnensis” in the “*Ca*. Korarchaeia” compared to any other lineages [6], possibly supporting our inference that methane metabolism may have evolved relatively early in *Thermoproteota*.

The *b*-type cytochrome in HdrDE complex belongs to the protein family of nitrate reductase gamma subunit (PF02665, NarI). Using all publicly available archaeal genomes, we found that the NarI domain-containing cytochromes (NarI-Cyt) are primarily used in three electron transfer complexes: HdrDE, dissimilatory nitrate reductase (NarGHI) [46], and sulfite reductase (DsrABCJKMOP). For the HdrDE and NarGHI complexes, the genes encoding the subunits are co-localized in archaeal genomes, each forming a transcriptional unit. However, in the Dsr complex, only a DsrK is co-localized with a DsrM (*b*-type cytochrome) while other subunits are usually not adjacent to the DsrKM but separated by few genes [6]. We examined distribution of the three complexes in archaea. A total of 101 genomes were found to encode these complexes (66 for HdrDE, 16 for Nar, 23 for Dsr), and they are distributed across the *Euryarchaeota* superphylum, *Thermoproteota*, and *Asgardarchaeota* (Supplementary Fig. 17 and Supplementary Table 9). Among these genomes, the HdrDE is found in methanogens and methanotrophs belonging to the class “*Ca*. Methanosarcinia”, the orders *Methanomicrobiales* and *Methanonatronarchaeales*, and in alkane-oxidizing archaea belonging to the orders *Archaeoglobales*, “*Ca*. Syntropharchaeales”, and *Methanosarcinales* (GoM-Arc1) (Supplementary Fig. 17). In Mcr-containing archaea outside of the *Euryarchaeota* superphylum, the complex is exclusively found in the “*Ca*. Methylarchaeales” (Fig. 1 and Supplementary Fig. 17).

**Fig. 5:**
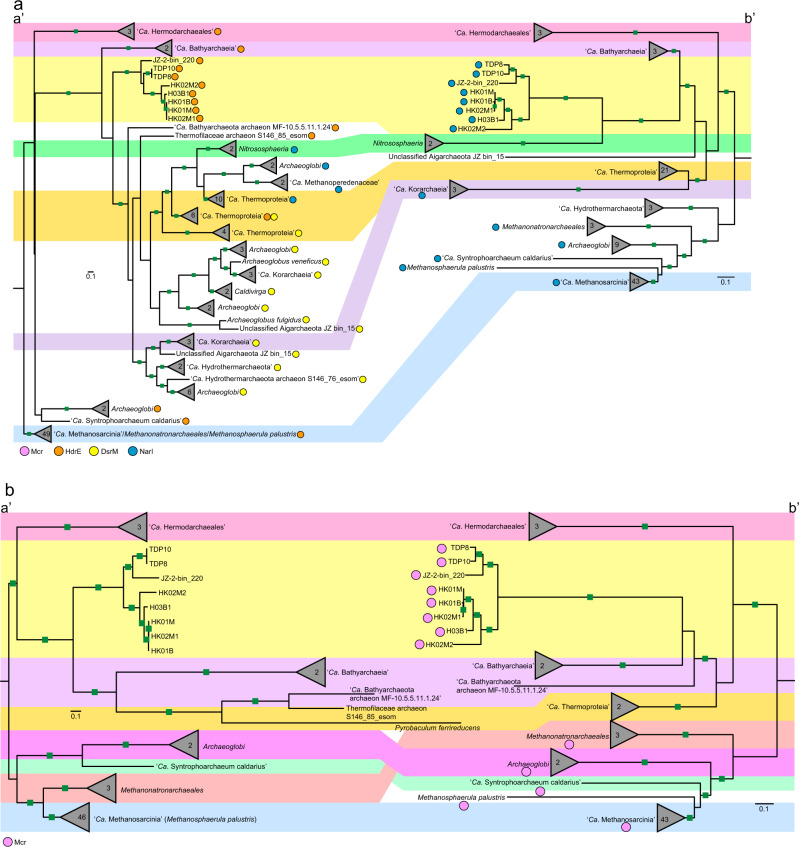
Phylogeny of NarI-domain-containing *b*-type cytochromes and concatenated HdrDE complexes in archaea. **a** Phylogeny of NarI-domain-containing *b*-type cytochromes in archaea. **b** Phylogeny of concatenated HdrDE complexes in archaea. The maximum-likelihood trees of NarI-domain-containing *b*-type cytochromes (**a** (a’)) and concatenated HdrDE subunits (**b** (a’)) from representative archaea are inferred with IQ-TREE (LG + C60 + F + G, -bb: 10,000 for NarI-domain, 1000 for HdrDE). The *b*-type cytochromes comprising different enzyme complexes are indicated by different color dots (light red for HdrE, yellow for DsrM, and blue for NarI). HdrE heterodisulfide reductase E subunit, DsrM sulfite reductase M subunit, NarI dissimilatory nitrate reductase I subunit. The maximum-likelihood trees of a concatenated set of 122 archaeal-specific marker proteins using the same genomes as those of NarI-domain-containing *b*-type cytochrome tree (**a** (b’)) and HdrDE complexe tree (**b** (b’)), respectively. The trees were computed with IQ-TREE using LG + C60 + F + G model. These genomes or clades with Mcr complexes are marked by pink dots. The bootstrap support values ≥95 are indicated with green filled squares.

Phylogenetic analyses of the NarI-Cyt were conducted to investigate the evolution of these genes in archaea (Fig. 5a). The results showed that these cytochromes have experienced frequent horizontal gene transfer, especially DsrM. The DsrM sequences annotated in members of the *Thermoproteota* form a distinct cluster. In the cluster, *Archaeoglobi* and “*Ca*. Hydrothermarchaeota” DsrM branch far from their *Euryarchaeota* superphylum relatives, and have potentially gained their cytochromes from a member of the “*Ca*. Korarchaeia”. Similarly, the “*Ca*. Methanoperedenaceae” and *Archaeoglobi* might have acquired their NarI genes from a member of *Thermoproteia*. Congruence between the cytochrome and genome-based trees for members of the *Thermoproteota* suggest that these cytochromes might have evolved before the diversification of this phylum. We further inferred a gene tree using concatenated HdrDE complex (Fig. 5b). The topological structure of this tree exhibits high congruence with the genome-based tree for all lineages except the *Methanonatronarchaeia*, supporting an early presence of the complex in archaea. This suggestion is supported by ALE analyses which indicate the presence of NarI-Cyt with high confidence in the common ancestor of *Thermoproteota* (pp = 0.69) and in the common ancestor of “*Ca*. Halobacteriota” (pp = 0.70) (Supplementary Fig. 17).

**Fig. 6:**
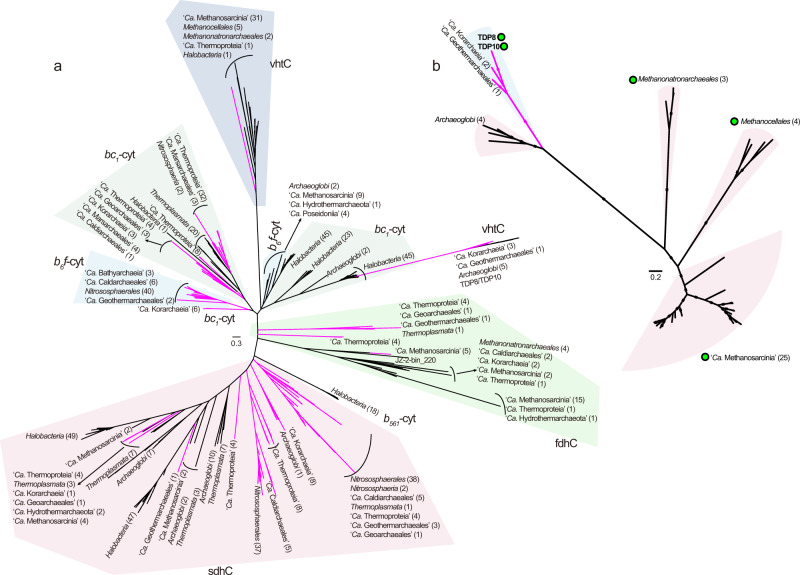
Phylogeny of *b*-type cytochromes and concatenated VhtAGC complexes in archaea. **a** Maximum-likelihood tree of *b*-type cytochromes of representative archaea (NarI-domain-containing *b*-type cytochromes not included) inferred using IQ-TREE (the best model: cpREV + F + G4). Different families of *b*-type cytochromes are shown. *vht* methanophenazine-reducing hydrogenase complex, *fdh* formate dehydrogenase, *sdh* succinate dehydrogenase. **b** Maximum-likelihood tree of concatenated VhtAGC subunits retrieved from representative archaea, inferred with IQ-TREE using LG + C60 + F + G model. The bootstrap support values ≥95 are indicated with filled squares. Genomes or clades with Mcr complexes are marked by green filled dots. The number of sequences for branches is given in parenthesis. The pink branches represent members of *Thermoproteota* phylum while the black branches represent members of *Euryarchaeota* superphylum.

As mentioned above, *b*-type cytochromes are classified into different protein families, and form part of many membrane-bound electron transfer complexes in bioenergetic pathways [47, 48]. Aside from HdrDE, Nar, and Dsr, such complexes also include Vht, Fdh, *b6f* complex, *bc1* complex, and succinate dehydrogenase (Sdh). We examined the distribution of different families of *b*-type cytochromes in 416 representative archaea covering 41 orders or phyla of the *Euryarchaeota* superphylum, *Thermoproteota*, and *Asgardarchaeota* (Supplementary Fig. 17 and Supplementary Table 9). A total of 246 genomes contained these *b*-type cytochromes that were distributed across 23 archaeal lineages. In total, 11 of the 13 lineages of the *Thermoproteota*, and 11 of the 24 orders in *Euryarchaeota* superphylum, had *b*-type cytochrome, suggesting its pervasiveness in archaea. We conducted phylogenetic analyses of the *b*-type cytochromes from different families (Fig. 6a). The result indicates that cytochromes from Fdh and Sdh complexes form two large clusters. Within each cluster, lineages from *Thermoproteota* or the *Euryarchaeota* superphylum were essentially grouped together, suggesting that these cytochromes may have evolved before the divergence of these major archaeal lineages. The cluster of cytochromes of the *b6f* complex is close to those of the *bc1* complex, consistent with the suggestion that bacterial cytochromes in *bc1* complex might originate from cytochromes in *b6f* complex [48]. A phylogenetic analysis of concatenated VhtAGC showed clustering of lineages from *Thermoproteota* with *Archaeoglobi* (Fig. 6b), suggesting ancient exchanges of the Vht complex among these lineages. Taken together, these results support an early origin of *b*-type cytochromes in archaea. Previous studies also imply that some core enzymes for bioenergetic pathways, including membrane-integral *b*-type cytochrome, formate dehydrogenase, [NiFe]-hydrogenase, the Rieske/cytb complexes, and NO-reductases, were present in the Last Universal Common Ancestor of Bacteria and Archaea [48, 49].

As the heme is indispensable to *b*-type cytochrome [47], we also investigated distribution of its biosynthetic pathway in archaea. Although there are 11 genes involving in the heme biosynthesis, the three genes (*Ahb-NirDH*, *Ahb-NirJ1*, and *Ahb-NirJ2*), responsible for conversion from precorrin-2 to heme, are the key to this pathway. Thus, these three genes were used as markers denoting the presence of heme biosynthetic pathway. Among 41 archaeal lineages, 32 had this pathway including the “*Ca*. Methylarchaeales” (Supplemental text, Fig. 2, Supplementary Fig. 17 and Supplementary Table 9). Phylogenetic analyses reveal that these lineages from *Thermoproteota* largely cluster together for *Ahb-NirDH* (Supplementary Fig. 18). However, for *Ahb-NirJ1* and *Ahb-NirJ2*, lineages from the *Euryarchaeota* superphylum, the *Thermoproteota*, and *Asgardarchaeota* are tangled up, suggesting frequent HGTs of these genes between these lineages. The wide distribution of this pathway across the *Euryarchaeota* superphylum, the *Thermoproteota*, and *Asgardarchaeota* (Supplementary Fig. 17 and Supplementary Table 9) suggests that a common ancestor may have been able to synthesize heme. This observation further supports the possibility of the early presence of *b*-type cytochromes in archaea.

Here we described the discovery of the novel archaeal order “*Ca*. Methylarchaeales”, expanding known methanogen and archaeal diversity. Members of the lineage are methyl-reducing methanogens that can conserve energy via membrane-bound electron transport chains. The “*Ca*. Methylarchaeales” are globally distributed in anoxic lake and marine sediments, suggesting that they make an important contribution to global methane emissions. Our broader analyses suggest that methanogens who use *b*-type cytochrome-containing complexes to transfer electrons may have originated before diversification of *Thermoproteota* or “*Ca*. Halobacteriota” phyla based on a conservative estimation for the origin of McrA and NarI-Cyt genes in the ALE analysis. A previous study using molecular clock analyses to indicate that the diversification of *Thermoproteota* likely occurred in the early Archean Eon [45]. Archean oceans are thought to have been anoxic and contain abundant ferrous iron from hydrothermal volcanics [50, 51], which would have provided sufficient raw materials for heme synthesis by methanogens. In addition, CO_2_, H_2_, and organic compounds produced by volcanic activity are transported to the early oceans [52], which provides adequate carbon and energy sources for methanogenic growth. Compared to hydrogenotrophic methanogens using electron bifurcation, methanogens using the membrane-bound electron chain have a higher energy production efficiency and growth yield, providing an advantage for members of the “*Ca*. Methylarchaeales” described here.

### Taxonomic proposals

#### “*Ca*. Methanotowutia igneaquae” (gen. nov., sp. nov.)

Methanotowutia (Me.tha.no.to.wu’ti.a. N.L. pref. methano-, pertaining to methane; N.L. fem. n. Methanotowutia methanogenic organism named after the lake Towuti in Indonesia where members of the genus were first discovered).

Methanotowutia igneaquae (ig.ne.a’quae. L. masc. adj. *igneus*, of fire; L. fem. n. aqua, freshwater, pertaining to freshwater habitats; N.L. gen. n. *igneaquae* from/of water of fire, referring to the volcanic lake environment). This organism is deduced to be able to use methylated compounds for methanogenesis. Representative genomes are near-complete bins TDP8 (Accession No. SAMN15658089) and TDP10 (Accession No. SAMN15658091) recovered from freshwater sediments in Lake Towuti in Indonesia with the latter the type genome for the species.

#### “*Ca*. Methanoinsularis halodrymi” (gen. nov., sp. nov.)

Methanoinsularis (Me.tha.no.in.su.la’ris. N.L. pref. methano-, pertaining to methane; L. fem. adj. *insularis*, from an island; N.L. fem. n. Methanoinsularis methanogenic organism from an island, specifically referring to Techeng Island in China where these microorganisms were discovered).

Methanoinsularis halodrymi (ha.lo.dry’mi. Gr. masc. n. hals (gen. *halos*) salt; Gr. masc. n. *drymos* coppice; N.L. gen. n. *halodrymi* of salty woodland, referring to the mangrove wetland environment). This uncultivated microorganism is assumed to be able to perform methylotrophic methanogenesis. The type genome for the species is the bin H03B1 (Accession No. SAMN15658086) recovered from mangrove wetlands in Techeng Island in China.

#### “*Ca*. Methanoinsularis haikouensis” (gen. nov., sp. nov.)

Methanoinsularis haikouensis (hai.kou.en’sis. N.L. fem. adj. haikouensis, pertaining to Haikou). This uncultivated microorganism is assumed to be able to perform methylotrophic methanogenesis. Representative genomes are the bins HK01M, HK01B, HK02M1 (Accession No. SAMN25131447, SAMN25131448, SAMN25131449) recovered from mangrove wetlands in Dongzhai Harbour in Haikou, China.

#### “*Ca*. Methanoporticola haikouensis” (gen. nov., sp. nov.)

Methanoporticola (Me.tha.no.por.ti’co.la. N.L. pref. methano-, pertaining to methane; L. masc. n. portus, harbour; L. suff. -cola (from L. masc. or fem. n. incola), inhabitant, dweller; N.L. masc. n. Methanoporticol, a methane-forming dweller of a harbor, specifically referring to Dongzhai Harbour in China where these microorganisms were discovered).

Methanoporticola haikouensis (hai.kou.en’sis. N.L. masc. adj. haikouensis, pertaining to Haikou). This uncultivated microorganism is assumed to be able to perform methylotrophic methanogenesis. The type genome for the species is the bin HK02M2 (Accession No. SAMN25131450) recovered from mangrove wetlands in Dongzhai Harbour in Haikou, China.

#### “*Ca*. Methylarchaeales” (ord. nov.)

Methylarchaeales (Me.thyl.ar.cha.ea’les. N.L. neut. n. Methylarchaeum (Candidatus) type genus of the order; -*ales*, ending denoting an order; N.L. fem. pl. n. Methylarchaeales, the order of the genus “*Ca*. Methylarchaeum”); Methylarchaeaceae (Me.thyl.ar.chae.a.ce’ae. N.L. neut. n. Methylarchaeum (Candidatus) type genus of the family); -*aceae*, ending denoting a family; N.L. fem. pl. n. Methylarchaeaceae, the family of the genus “*Ca*. Methylarchaeum”).

## Materials and methods

### Sample collection and DNA sequencing

Thirteen sediment samples were obtained from mangrove wetlands on Techeng Island, Zhanjiang, Guangdong, China on November 25, 2018, and in Dongzhai Harbour, Haikou, China on September 30, 2021 (Supplementary Fig. 1). In each wetland, the two to three cores (1 m deep and 2–10 m apart) were taken using a peat sampler (two cores for Techeng Island; three cores for Dongzhai Harbour). Each sediment core was evenly divided into three parts in an anoxic glove box. Sediments from subsurface (15–20 cm depth), middle (40–45 cm depth), and bottom (95–100 cm depth) layers were put into plastic bags immediately after collection, kept in a sampling box with dry ice, transported to the laboratory and stored at −80 °C for further analysis. The detailed sampling information is shown in Supplementary Fig. 1.

Genomic DNA was extracted from ~10 g of sediment samples with the PowerSoil DNA Isolation Kit (MoBio Laboratories, Carlsbad, CA, USA). Metagenomic sequencing was conducted on HiSeq 2500 platform (Illumina, San Diego, CA, USA) at Guangdong MagiGene Technology Company (Guangzhou, China). Each sample from Techeng Island wetland generated about 60 Gbp of raw sequence data (2 × 150 bp paired-end reads), while 100 Gbp of sequencing data per sample were obtained for mangrove sediment from Dongzhai harbour.

### Genome assembly and binning

Raw reads generated from mangrove wetland sediments were quality filtered and pruned using Trimmomatic [53]. The resulting clean reads were assembled using MEGAHIT [54] with the following parameters: --presets meta-large, --min-contig-len 500 and using IDBA-UD [55] with the following parameters: -mink 55, -maxk 105, -steps 10, --min_contig 500, --pre_correction, respectively. The contigs/scaffolds generated were binned using MetaBat2 [56] 8 times, with 8 combinations of specificity and sensitivity parameters (-m 1500, --maxP 95 or 60, --minS 60 or 95; --maxEdges 200 or 500). All binning results were merged and refined using DAS Tool [57] (--score threshold 0.25, v1.1.1). Contigs or scaffolds within these bins with divergent GC content or tetranucleotide signatures or coverage profiles were removed with mmgenome [58] and RefineM [59]. The resulting bins were refined manually to remove contaminating contigs/scaffolds based on multi-copy marker genes. The local assembly errors for contigs/scaffolds were checked using ra2.py (https://github.com/christophertbrown/fix_assembly_errors/blob/master/ctbRA/ra2.py) [60]. CheckM [61] was used to assess completeness, contamination, and strain heterogeneity. Finally, six MAGs (H03B1, H03B2, HK01M, HK01B, HK02M1, and HK02M2) containing an *mcrA* gene, representing high-quality genomes based on genome reporting standards [62], were obtained from these metagenomic dataset. H03B1 and H03B2 were generated from the same sample using different assembling tools (MEGAHIT for H03B1; IDBA-UD for H03B2) (Supplementary Table 10). They had a 99.8% average nucleotide identity (ANI) to each other, possibly representing the same strain [63]. As the H03B1 had a higher estimated completeness, it was used for further analysis.

Two metagenomic datasets generated from sediments in Lake Towuti, South Sulawesi, Indonesia were transformed to FASTQ file with Fastq-dump using --split-3 (https://ncbi.github.io/sra-tools/fastq-dump.html) and then processed with Trimmomatic [53]. Processed reads were assembled using MEGAHIT [54] (--presets meta-large, --min-contig-len 500), and using IDBA-UD [55] (-mink 34, -maxk 124, -steps 10, --min_contig 500, --pre_correction), respectively. Binning of generated contigs/scaffolds, and genomic curation and refining steps were performed following the procedures as described above. As a result, four *mcrA*-containing genomic bins (TDP7-10) were obtained (Supplementary Table 10). The four *mcrA* gene sequences were identical to one another (100% aa identity), and had high sequence similarity to the H03B1 *mcrA* genes (87.6% aa identity). TDP7 and TDP8 genomes were obtained from TDP7 metagenome with MEGAHIT [54] and IDBA-UD [55], respectively while TDP9 and TDP10 genomes were produced from TDP9 metagenome using MEGAHIT [54] and IDBA-UD [55], respectively. TDP7 and TDP8, and TDP9 and TDP10 had a high nucleotide sequence similarity one another (98.1% and 97.5% ANI, respectively), probably representing the same strain [63]. The TDP8 and TDP10 bins were selected to represent these MAGs in further analyses given their higher completeness estimates.

### Concatenated ribosomal RNA gene tree phylogeny

The 16S and 23S rRNA genes of the “*Ca*. Methylarchaeales” bins were predicted with Barrnap (https://github.com/tseemann/barrnap). Four 16S rRNA gene and five 23S rRNA sequences were identified in these MAGs (Supplementary Table 4). Reference 16S and 23S rRNA gene sequences that were derived from 145 genomes, representing the diversity of the *Thermoproteota* phylum, were used to infer a phylogenetic tree. The 16S and 23S rRNA sequences from reference genomes of *Halobacteria* were used as the outgroup. All 16S and 23S rRNA gene sequences were aligned with MAFFT (--auto) [64], pruned with BMGE [65] (-m DNAPAM250:4 -g 0.5) and concatenated. The topology of maximum-likelihood trees were computed with IQ-TREE [66] using the command: “-m TEST (GTR + F + I + G4), -bb 1000”. Trees were edited using iTOL [67] and modified using Adobe Illustrator.

### Concatenated marker gene tree phylogeny

A set of representative good-quality archaeal genomes consisting of 419 taxa which covered currently known archaeal lineages were used in the genome trees (Supplementary Table 9). The trees were inferred using a concatenated set of 122 archaeal-specific single copy marker genes in the GTDB (https://gtdb.ecogenomic.org/) (Supplementary Table 11). The orthologs of these marker genes in the “*Ca*. Methylarchaeales” MAGs and the reference genomes were identified using GTDB-Tk tool [20] (v1.3.0, https://github.com/Ecogenomics/GTDBTk) based on hidden Markov models. Maximum-likelihood trees were constructed with IQ-TREE [66] using the following command: “-m LG + C60 + F + G, -bb 1000”. Trees were edited using iTOL [67], using the DPANN superphylum as an outgroup, and modified using Adobe Illustrator.

### Genome annotation and metabolic analysis

Gene prediction was conducted with Prodigal [68] using -p meta. Functional protein annotation was carried out by searching against arCOGs and nr database with BLASTP [69] (e-value <1e−5). Pfam database and InterproScan [70] were used to further analyze protein function. KEGG database [71] was used as reference to reconstruct metabolic pathways. The *mcrABCDG* genes were confirmed by searching against *mcrABCDG* genes from Pfam with HMMER [72] (Supplementary Table 12). Carbohydrate enzymes were annotated on dbCAN webserver [73], and peptidases were identified using eggNOG-mapper and verified with comparisons against nr annotations. Subcellular localization of carbohydrate enzymes and peptidases were predicted using CELLO (v.2.5) [74]. The motifs and active sites of McrA, HdrD, Fpo-like, and *b*-type cytochromes (NarI-Cyt, VhtC, and FdhC) were analyzed according to previous studies [15, 25, 36, 75]. Transmembrane helices of *b*-type cytochromes were analyzed with TMHHMM Server (v. 2.0) (http://www.cbs.dtu.dk/services/TMHMM/).

### Functional gene phylogeny and gene tree-species tree reconciliation

#### Phylogenies of McrABG

The *mcrABG* genes from reference genomes (Supplementary Table 9) were identified by searching against arCOGs using BLASTP [69], and then confirmed by searching against *mcrABG* genes from Pfam using HMMER [72]. MAFFT (--auto) [64] and IQ-TREE [66] were used for sequence alignment and construction of phylogenetic trees, respectively. The model used in IQ-TREE was LG + C60 + F + G for concatenated *mcrABG* genes, while it was LG + F + I + G4 for *mcrA* and *mcrB* genes, and LG + G4 for *mcrG* gene. Ultrafast bootstrapping (1000 replicates) was adopted for these trees.

#### Phylogeny of six concatenated methanogenesis markers (m4–m9)

The six conserved markers were retrieved according to arCOGs accession number provided by a previous study [44] in the “*Ca*. Methylarchaeales” MAGs and *mcr*-containing genomes available in the NCBI or IMG-databases. These sequences were aligned with MAFFT (--auto), trimmed with BMGE (-m BLOSUM30 -b 3 -g 0.5), and concatenated. Before concatenation, maximum-likelihood trees for each gene were computed with IQ-TREE (-m TEST, -bb 1000) for inspection of congruence. Genes that lead to intense incongruences at order or phylum level (bootstrap value ≥80%) were discarded. Maximum-likelihood phylogeny of concatenated markers was inferred using IQ-TREE (LG + C60 + F + G, -bb 1000).

#### Phylogenies of b-type cytochromes and concatenated HdrDE

The *b*-type cytochrome genes from the “*Ca*. Methylarchaeales” and 408 representative genomes from *Euryarchaeota* superphylum, *Thermoproteota*, and *Asgardarchaeota* were identified by searching all predicted genes in a genome against custom hmm profiles for NarI-Cyt, prokaryotic cytochrome *b*_*561*_, succinate dehydrogenase cytochrome B small subunit, cytochrome *bc*_*1*_ complex subunit 8, cytochrome *b*_*6*_*f* complex subunit VI (PetL), Cytochrome *b/b*_*6*_*/petB*, Ni/Fe-hydrogenase *b*-type cytochrome subunit, succinate dehydrogenase cytochrome *b*_*556*_, cytochrome *b*_*558/566*_ subunit B, and cyt *b*_*6*_*/f* complex subunit IV using HMMER. Hits were confirmed by comparing with arCOGs and nr annotations. For NarI-Cyt, it was manually verified to ensure that it is collocated with a gene encoding HdrD, or DsrK or NarGH in a genome. Owing to the lack of sequence similarity between NarI-Cyt and other *b*-type cytochromes, the phylogenetic tree of NarI-Cyt was built independently. Sequences were aligned by MAFFT (--auto) [64]. Maximum-likelihood trees were constructed using IQ-TREE [66] (the model: LG + C60 + F + G for NarI-Cyt, cpREV+F + G4 for other *b*-type cytochromes, -bb 1000). The HdrD and HdrE was concatenated and its phylogeny was inferred in IQ-TREE (LG + C60 + F + G, -bb 1000).

#### Phylogeny of concatenated VhtACG

VhtACG genes from the “*Ca*. Methylarchaeales” and 408 representative archaeal genomes were identified by searching all predicted genes in a genome against VhtA, VhtC, and VhtG hmm databases from Pfam with HMMER, and confirmed with comparisons against arCOGs and nr annotations. Furthermore, gene arrangement was checked to ensure that the VhtAGC subunit genes are collocated in a genome. These sequences were aligned using MAFFT (--auto), trimmed with trimAl [76] (-automated1), and concatenated. Before concatenation, maximum-likelihood tree of each subunit was constructed with IQ-TREE for checking of congruence. No strong incongruences were found. Maximum-likelihood trees were computed with IQ-TREE [66] (the model: LG + C60 + F + G).

#### Phylogenies of MtrH, group 4 [NiFe] hydrogenases and geranylfarnesyl diphosphate synthase

For MtrH, homologs from the “*Ca*. Methylarchaeales” were identified by searching against arCOGs and nr database using BLASTP. Reference sequences were derived from a previous study [24]. For group 4 [NiFe] hydrogenases, catalytic subunit of group 4 [NiFe] hydrogenases homologs from the “*Ca*. Methylarchaeales” were identified using arCOGs, and confirmed with HydDB [77]. Reference sequences were downloaded from HydDB. For geranylfarnesyl diphosphate synthase, homologs from the “*Ca*. Methylarchaeales” were annotated with arCOGs while reference sequences refer to a previous study [31]. Sequences were aligned using MAFFT (--auto) and trimmed with BMGE (-m BLOSUM30 -b 3 -g 0.9). IQ-TREE (-m TEST, -bb 1000) was used to infer these trees.

#### Phylogenies of the key genes for the heme biosynthesis pathway (Ahb-NirDH, Ahb-NirJ1 and Ahb-NirJ2)

These genes from the “*Ca*. Methylarchaeales” and 408 representative archaeal genomes were identified using eggNOG-mapper. Hits were confirmed by searching against arCOGs and nr databases using BLASTP. Sequences were aligned using MAFFT (--auto) and trimmed with trimAl (-automated1). Maximum-likelihood trees were constructed with IQ-TREE (-m TEST, -bb 1000).

#### Gene tree-species tree reconciliation

The ALE analyses were performed using the ALEml_undated algorithm of the ALE package [78] (v1.0, https://github.com/ssolo/ALE). A sample of 1000 and 10,000 trees that were produced in IQ-TREE (-bb: 1000 for McrA, 10,000 for NarI-Cyt) for each gene family were reconciled with their rooted species trees. The presence probability of gene family as well as duplication, transfer and loss events were estimated in each internal node in the rooted species tree.

## Supplementary information

Supplementary Materials

Supplementary Table 1

Supplementary Table 2

Supplementary Table 3

Supplementary Table 5

Supplementary Table 6

Supplementary Table 7

Supplementary Table 9

Supplementary Table 12

Dataset 1

## Data Availability

Genomes are archived in the NCBI database under BioProject ID PRJNA648665. Genome bins can be found at NCBI under the Accession numbers SAMN15658086 (H03B1), SAMN15658087 (H03B2), SAMN25131447 (HK01M), SAMN25131448 (HK01B), SAMN25131449 (HK02M1), SAMN25131450 (HK02M2), SAMN15658088 (TDP7), SAMN15658089 (TDP8), SAMN15658090 (TDP9), SAMN15658091 (TDP10). Related raw reads have been submitted to Sequence Read Archive under SRA accession PRJNA629047.
