# Supplementary material for: Expanding the phylogenetic distribution of cytochrome b-containing methanogenic archaea sheds light on the evolution of methanogenesis
Source: ISME J. 2022 Jul 9;16(10):2373–87. doi: 10.1038/s41396-022-01281-0 (PMC9478090; doi:10.1038/s41396-022-01281-0)
Supplement: Supplementary file 1 — Supplementary Materials [file 41396_2022_1281_MOESM1_ESM.pdf]

Supplementary Information:

Expanding the phylogenetic distribution of cytochrome *b*-containing methanogenic archaea sheds light on the evolution of methanogenesis

Ya-Fei Ou[1]†, Hong-Po Dong[1]*†, Simon J. Mcllroy[2], Sean A. Crowe[3,4], Steven J. Hallam[4], Ping Han[5], Jens Kallmeyer[6], Rachel L. Simister[3,4], Aurele Vuillemin[6], Andy O. Leu[2], Zhanfei Liu[7], Yan-Ling Zheng[5], Qian-Li Sun[1], Min Liu[5], Gene W. Tyson[2], Li-Jun Hou[1]*

[1]State Key Laboratory of Estuarine and Coastal Research, East China Normal University, Shanghai 200241, China; [2]Centre for Microbiome Research, School of Biomedical Sciences, Queensland University of Technology (QUT), Translational Research Institute, Queensland 4102, Australia; [3]Ecosystem Services, Commercialization Platforms, and Entrepreneurship (ECOSCOPE) Training Program, University of British Columbia, Vancouver, British Columbia, Canada; [4]Department of Microbiology & Immunology, University of British Columbia, Vancouver, British Columbia, Canada; [5]Key Laboratory of Geographic Information Science, Ministry of Education, East China Normal University, Shanghai 200241, China; [6]GFZ German Research Centre for Geosciences, Helmholtz Centre Potsdam, Potsdam, Germany; [7]Marine Science Institute, The University of Texas at Austin, Port Aransas, TX 78373, USA.

Supplementary text

Mcr complex tertiary structure

Conformation of the H03B1 McrABG subunits were analyzed with I-TASSER [1] v. 5.1 using the default setting. For McrA, McrB, and McrG, the displayed structural models (Supplementary Fig. 13b) were overlaid on the *Methanosarcina barkeri* Mcr crystal structure (PDB ID: 1e6y). The structural alignment TM-scores for McrA, McrB, and McrG were 0.980, 0.964, and 0.930, respectively, while the RMSDs of the TM-aligned residues were 0.56, 0.97, and 0.99 Å for McrA, McrB, and McrG, respectively. The binding sites for CoM, CoB, and F_{430} in McrA were analyzed using COACH package in I-TASSER, showing that they were identical between H03B1 and the *Methanosarcina barkeri* Mcr crystal structure (Supplementary Fig. 13c).

Vertical distribution of the '*Ca.* Methylarchaeales' in sampling sites

To probe vertical distribution of the '*Ca.* Methylarchaeales' MAGs in sediment cores of sampling sites, the H03B1 genome was used to recruit reads from six metagenomes generated from Techeng Island while HK01M, HK01B, HK02M1, and HK02M2 recruit reads from five metagenomes generated from Dongzhai Harbour. Metagenomic reads were mapped to these MAGs using Bowtie2 (v.2.3.5; -no-unal) [2]. The resulting SAM files were converted to BAM files with samtools [3]. CoverM (https://github.com/wwood/CoverM) was used to screen for reads with ≥ 95% identity and ≥ 75% alignment length. Read counts were used to compute reads per kbp of each genome per Mbp of each metagenome (RPKM; (reads recruited to a genome/(length of genome in bp/1,000))/(total bp in metagenome/1,000,000)).

In total, relative abundance of these MAGs appeared to increase gradually with depth (Supplementary Fig. 4). They were very rare at the 15 cm depth below the surface (RPKM: 0.0001-0.0039). The highest values were observed at the 100 cm depth (PPKM: 0.0090-0.1726). This profile may be related to oxygen distribution in sediment. It has been reported that mangrove wetlands have high density of crab burrows, some of which can reach 30-40 cm depth [4]. By these burrows, oxygen is able to penetrate deep layers of sediment. In addition, the roots of mangrove plants

also can release substantial amounts of oxygen into the rhizosphere and affect biogeochemical processes around the roots. Based on the analyses, it is inferred that the '*Ca.* Methylarchaeales' may be highly sensitive to oxygen. A previous study revealed that in most mangrove wetlands including our sampling sites, soil organic carbon content tended to reduce with increasing depth. The highest organic carbon content was observed in the upper fractions (0-10 cm and 10-20 cm) [5]. Thus, it seems that the abundance of the '*Ca.* Methylarchaeales' is inversely correlated with the organic carbon content.

Environmental distribution of the '*Ca.* Methylarchaeales'

In order to investigate distribution of the '*Ca.* Methylarchaeales' in the environment, the 16S rRNA and *mcrA* genes from the '*Ca.* Methylarchaeales' were used to screen for homologs across public sequence databases. Over 300 public metagenome datasets of non-human were obtained from the SRA database (https://www.ncbi.nlm.nih.gov/sra/?term=) and IMG/M database (https://img.jgi.doe.gov/cgi-bin/m/main.cgi). The SRA files were transformed to FASTQ with SRA Toolkit (https://trace.ncbi.nlm.nih.gov/Traces/sra/sra.cgi?view=software). Reads in each metagenome were mapped to16S rRNA genes of the Silva v138.1 NR99 dereplicated database [6] and the '*Ca.* Methylarchaeales' using BWA-MEM [7] with default parameters, and converted to a sorted indexed BAM file using samtools [3]. The BAM file was filtered using CoverM (https://github.com/wwood/CoverM) with filter mode to only retain hits with ≥ 97% identity and ≥ 50% alignment length to a 16S rRNA gene. Reads mapping to the '*Ca.* Methylarchaeales' 16S rRNA genes were identified in the BAM file (Supplementary Table 3), and the percent coverage was calculated using samtools [3] with coverage mode. The highest percent alignment of the reference '*Ca.* Methylarchaeales' 16S rRNA genes was reported (Supplementary Table 3). For screening using the *mcrA* gene, reads in metagenomes were mapped to a dataset of 196 *mcrA* genes (including *Thermoproteota*, *Asgardarchaeota* and *Euryarchaeota* superphylum *mcrA*) using BWA-MEM with default parameters. Then, the sorted and indexed BAM file was filtered using CoverM with filer mode. Reads

were counted if they met the defined cutoff values (≥ 85% nucleotide identity and ≥ 50% alignment length) and reported for the '*Ca.* Methylarchaeales' *mcrA* (Supplementary Table 3). Percent coverage of *mcrA* genes was computed as detailed for the 16S rRNA gene analysis.

Screening of the Silva 16S rRNA sequence database [6] identified two genes highly similar to 16S rRNA gene of the H03B1 MAG that were generated from anoxic, sulfide-rich bottom water of Lake Vilar (Spain) and likely represent members of the same family (AJ937875 and AJ937878, > 91% identity; > 89% alignment length) [8]. Searching of NCBI nr database did not find close homologs of the '*Ca.* Methylarchaeales' McrA (Top hit is McrA of member of '*Ca.* Korarchaeia', 74% aa identity). In addition, by mapping reads against a *mcrA* database and a 16S rRNA database, respectively, reads that were highly similar to the '*Ca.* Methylarchaeales' *mcrA* genes (≥ 85% nucleotide identity; ≥ 50% alignment length) and the 16S rRNA genes (≥ 97% identity; ≥ 50% alignment length) were found in three metagenomes derived from mangrove wetlands and 10 metagenomes generated from sediments of Lake Towuti, Lake Matano and Gulf of Boni in Indonesia (Supplementary Table 3; Supplementary Fig. 5). These reads provided almost full-length alignment of the '*Ca.* Methylarchaeales' *mcrA* genes (71.3-100%) and 16S rRNA genes (85.4-100%) (Supplementary Fig. 5a and b). The reads mapping to '*Ca.* Methylarchaeales' *mcrA* genes were assembled using MEGAHIT, and the resulting McrA fragments clustered with '*Ca.* Methylarchaeales' McrA sequences in phylogenetic tree (Supplementary Fig. 5c). Screening of metagenomes from marine sediments enriched in hydrocarbon compounds (deep-sea hydrothermal vents, petroleum seep), hot springs, mangrove wetlands, palm oil mill effluent, and rice paddy soils (Supplementary Table 3) also identified samples with positive hits to the '*Ca.* Methylarchaeales' 16S rRNA gene but not the *mcrA* gene for this lineage. This is likely due to the low abundance of members of this lineage in these samples.

Heme biosynthetic pathway of the '*Ca.* Methylarchaeales'

Given that all cytochromes use hemes as cofactors, we examined pathway of heme biosynthesis in '*Ca.* Methylarchaeales'. H03B1, HK01M, HK02M1, HK02M2,

TDP8, and TDP10 contained an almost complete set of genes encoding the archaeal pathway of heme biosynthesis (Fig. 2 and Supplementary Table 7). In this pathway, glutamyl-tRNA is first converted to uroporphyrinogen III by the five enzymes (HemA, HemL, HemB, HemC, and HemD) which is the common precursor in heme biosynthesis of all three domains of life [9]. And then uroporphyrinogen III is further transformed into heme via the five archaea-specific enzymes (SUMT, PC2-DH/sirB, Ahb-NirDH, Ahb-NirJ1, and Ahb-NirJ2), as previously reported in *M. barkeri* [10]. However, '*Ca.* M. tengchongensis' lacked most of genes in the heme biosynthesis pathway. It is unclear if these genes are in missing region of the genome or that an unknown pathway is responsible for heme synthesis. These results further support the presence of cytochromes involved in electron transport chain in '*Ca.* Methanoinsularis', '*Ca.* Methanoporticola' and '*Ca.* Methanotowutia'.

Supplementary Table 4. Characteristics of 16S rRNA and 23S rRNA genes in the '*Ca.* Methylarchaeales' bins.

	MAGs	H03B1	H03B2	TDP8	TDP10	HK01M	HK01B
16S rRNA	Gene length (bp)	773	1500	803	913	-	-
	Contig/Scaffold length (bp)	21597	50587	5479	31969	-	-
	Start position (bp)	0	17824	4674	3658	-	-
	End position (bp)	773	19324	5477	4571	-	-
23S rRNA	Gene length (bp)	1041	3034	-	1193	3037	1019
	Contig/Scaffold length (bp)	29149	50587	-	31969	19492	4339
	Start position (bp)	0	19476	-	1	16245	1
	End position (bp)	1041	22510	-	1194	19281	1019

Supplementary Table 8. Conserved active sites of methyl-coenzyme M reductase subunit alpha identified by Ermler et al [11].

Residues	Proposed Function	*Eurarchaeota* superphylum	‘*Ca.* Bathy-archaeia’	‘*Ca.* Kor-archaeia’	‘*Ca.* Nezha-archaeales’	‘*Ca.* Methano-methylicales’	‘*Ca.* Methylarchaeales’ (This study)
α‘147	F430 axial ligand	Gln	Gln	Gln	Gln	Gln	Gln
α’225	CoB interacting	Arg	Arg	Arg	Arg	Arg	Arg
α’256	CoB interacting	Lys	Lys	Lys	Lys	Lys	Lys
α’257	CoB interacting, methylated	His	His	His	His	His	His
α270	CoB interacting	Arg	**Gln**	Arg	Arg	Arg	Arg
α271	Methylated	Arg	Arg	Arg	Arg	Arg	Arg
α330	Substrate cavity wall	Phe	Phe	Phe	Phe	Phe	Phe
α333	COM interacting	Tyr	**Thr**	Tyr	Tyr	Tyr	Tyr
α400	Methylated	Gln	**His**	Gln	Gln	Gln	Gln
α443	COM interacting	Phe	**Trp**	Phe	Phe	Phe	Phe
α445	Thiol substituted	Gly	Gly	Gly	Gly	Gly	Gly
α452	Methylated	Cys	**Ile**	**Met**	Cys	Cys	**Ala/Ser**
α481	COB interacting	Asn	**Thr/Ser**	Asn	Asn	Asn	Asn

Supplementary Table 10. Genome characteristics of the '*Ca.* Methylarchaeales' bins obtained using different assembly methods.

**Samples**	**H03B**		**TDP7**		**TDP9**	
**MAGs**	H03B1	H03B2	TDP7	TDP8	TDP9	TDP10
**AAI/ANI**	99.84/99.79		98.31/98.07		97.95/97.49	
**Assembling tool**	MEGAHIT	IDBA_UD	MEGAHIT	IDBA_UD	MEGAHIT	IDBA_UD
**Binning Method**	Metabat2	Metabat2	Metabat2	Metabat2	Metabat2	Metabat2
**Length (Mbp)**	1.46	1.48	2.32	2.55	2.08	2.45
**Completeness (%)**	96.60	93.69	91.75	94.69	90.42	99.51
**Contamination (%)**	0.97	0.97	1.94	0.97	0.00	0.97
**N50 (bp)**	25415	19579	5010	5614	14046	53503
**Number of contigs/scaffolds**	86	118	548	570	247	141
**GC content (%)**	42.51	42.45	38.57	38.78	38.87	38.95
**16S rRNA**	Yes	Yes		Yes		Yes
mcrABCDG	Yes	Yes	Yes	Yes	Yes	Yes
hdrDE	Yes	Yes	Yes	Yes	Yes	Yes
vhtAGC			Yes	Yes	Yes	Yes
**Wood–Ljungdahl pathway**	Yes	Yes	Yes	Yes	Yes	Yes

Supplementary Table 11. List of 122 archaeal-specific conserved marker genes from GTDB-Tk [12] used for phylogenetic inference.

Marker ID	Name	Description
PF01990.12	ATP-synt_F	ATP synthase (F/14-kDa) subunit
PF01866.12	Diphthamide_syn	Putative diphthamide synthesis protein
PF04104.9	DNA_primase_lrg	Eukaryotic and archaeal DNA primase, large subunit
PF01984.15	dsDNA_bind	Double-stranded DNA-binding domain
PF02006.11	DUF137	Protein of unknown function DUF137
PF04019.7	DUF359	Protein of unknown function (DUF359)
PF01864.12	DUF46	Putative integral membrane protein DUF46
PF04919.7	DUF655	Protein of unknown function (DUF655)
PF07541.7	EIF_2_alpha	Eukaryotic translation initiation factor 2 alpha subunit
PF13685.1	Fe-ADH_2	Iron-containing alcohol dehydrogenase
PF01269.12	Fibrillarin	Fibrillarin
PF00368.13	HMG-CoA_red	Hydroxymethylglutaryl-coenzyme A reductase
PF01798.13	Nop	Putative snoRNA binding domain
PF00687.16	Ribosomal_L1	Ribosomal protein L1p/L10e family
PF00466.15	Ribosomal_L10	Ribosomal protein L10
PF00827.12	Ribosomal_L15e	Ribosomal L15
PF01280.15	Ribosomal_L19e	Ribosomal protein L19e
PF01157.13	Ribosomal_L21e	Ribosomal protein L21e
PF01198.14	Ribosomal_L31e	Ribosomal protein L31e
PF01655.13	Ribosomal_L32e	Ribosomal protein L32
PF01090.14	Ribosomal_S19e	Ribosomal protein S19e
PF01282.14	Ribosomal_S24e	Ribosomal protein S24e
PF01200.13	Ribosomal_S28e	Ribosomal protein S28e
PF01015.13	Ribosomal_S3Ae	Ribosomal S3Ae family
PF00900.15	Ribosomal_S4e	Ribosomal family S4e
PF01092.14	Ribosomal_S6e	Ribosomal protein S6e
PF00410.14	Ribosomal_S8	Ribosomal protein S8
PF01000.21	RNA_pol_A_bac	RNA polymerase Rpb3/RpoA insert domain
PF13656.1	RNA_pol_L_2	RNA polymerase Rpb3/Rpb11 dimerisation domain
PF01194.12	RNA_pol_N	RNA polymerases N / 8 kDa subunit
PF03874.11	RNA_pol_Rpb4	RNA polymerase Rpb4
PF01191.14	RNA_pol_Rpb5_C	RNA polymerase Rpb5, C-terminal domain
PF02978.14	SRP_SPB	Signal peptide binding domain
PF01868.11	UPF0086	Domain of unknown function UPF0086
PF01496.14	V_ATPase_I	V-type ATPase 116kDa subunit family
TIGR00021	rpiA	ribose 5-phosphate isomerase A
TIGR00037	eIF_5A	translation elongation factor IF5A
TIGR00042	TIGR00042	non-canonical purine NTP pyrophosphatase, RdgB/HAM1 family
TIGR00064	ftsY	signal recognition particle-docking protein FtsY
TIGR00111	pelota	mRNA surveillance protein pelota
TIGR00134	gatE_arch	glutamyl-tRNA(Gln) amidotransferase, subunit E
TIGR00240	ATCase_reg	aspartate carbamoyltransferase, regulatory subunit
TIGR00264	TIGR00264	alpha-NAC homolog
TIGR00270	TIGR00270	TIGR00270 family protein
TIGR00279	uL16_euk_arch	ribosomal protein uL16
TIGR00283	arch_pth2	peptidyl-tRNA hydrolase
TIGR00291	RNA_SBDS	rRNA metabolism protein, SBDS family
TIGR00293	TIGR00293	prefoldin, alpha subunit
TIGR00307	eS8	ribosomal protein eS8
TIGR00308	TRM1	N2,N2-dimethylguanosine tRNA methyltransferase

TIGR00323	eIF-6	putative translation initiation factor eIF-6
TIGR00324	endA	tRNA-intron lyase
TIGR00335	primase_sml	putative DNA primase, eukaryotic-type, small subunit
TIGR00336	pyrE	orotate phosphoribosyltransferase
TIGR00337	PyrG	CTP synthase
TIGR00373	TIGR00373	transcription factor E
TIGR00389	glyS_dimeric	glycine--tRNA ligase
TIGR00392	ileS	isoleucine--tRNA ligase
TIGR00398	metG	methionine--tRNA ligase
TIGR00405	KOW_elon_Spt5	transcription elongation factor Spt5
TIGR00408	proS_fam_I	proline--tRNA ligase
TIGR00422	valS	valine--tRNA ligase
TIGR00425	CBF5	putative rRNA pseudouridine synthase
TIGR00432	arcsn_tRNA_tgt	tRNA-guanine(15) transglycosylase
TIGR00442	hisS	histidine--tRNA ligase
TIGR00448	rpoE	DNA-directed RNA polymerase
TIGR00456	argS	arginine--tRNA ligase
TIGR00458	aspS_nondisc	aspartate--tRNA(Asn) ligase
TIGR00463	gltX_arch	glutamate--tRNA ligase
TIGR00468	pheS	phenylalanine--tRNA ligase, alpha subunit
TIGR00471	pheT_arch	phenylalanine--tRNA ligase, beta subunit
TIGR00490	aEF-2	translation elongation factor aEF-2
TIGR00491	aIF-2	translation initiation factor aIF-2
TIGR00501	met_pdase_II	methionine aminopeptidase, type II
TIGR00521	coaBC_dfp	phosphopantothenoylcysteine decarboxylase /phosphopantothenate--cysteine ligase
TIGR00522	dph5	diphthine synthase
TIGR00549	mevalon_kin	mevalonate kinase
TIGR00658	orni_carb_tr	ornithine carbamoyltransferase
TIGR00670	asp_carb_tr	aspartate carbamoyltransferase
TIGR00729	TIGR00729	ribonuclease HII
TIGR00936	ahcY	adenosylhomocysteinase
TIGR00982	uS12_E_A	ribosomal protein uS12
TIGR01008	uS3_euk_arch	ribosomal protein uS3
TIGR01012	uS2_euk_arch	ribosomal protein uS2
TIGR01018	uS4_arch	ribosomal protein uS4
TIGR01020	uS5_euk_arch	ribosomal protein uS5
TIGR01025	uS19_arch	ribosomal protein uS19
TIGR01028	uS7_euk_arch	ribosomal protein uS7
TIGR01038	uL22_arch_euk	ribosomal protein uL22
TIGR01046	uS10_euk_arch	ribosomal protein uS10
TIGR01052	top6b	DNA topoisomerase VI, B subunit
TIGR01060	eno	phosphopyruvate hydratase
TIGR01077	L13_A_E	ribosomal protein uL13
TIGR01080	rplX_A_E	ribosomal protein uL24
TIGR01213	pseudo_Pus10arc	tRNA pseudouridine(54/55) synthase
TIGR01309	uL30_arch	ribosomal protein uL30
TIGR01952	nusA_arch	NusA family KH domain protein, archaeal
TIGR02076	pyrH_arch	putative uridylate kinase
TIGR02153	gatD_arch	glutamyl-tRNA(Gln) amidotransferase, subunit D
TIGR02236	recomb_radA	DNA repair and recombination protein RadA
TIGR02258	2_5_ligase	2'-5' RNA ligase
TIGR02338	gimC_beta	prefoldin, beta subunit
TIGR02389	RNA_pol_rpoA2	DNA-directed RNA polymerase, subunit A"
TIGR02390	RNA_pol_rpoA1	DNA-directed RNA polymerase subunit A'

TIGR02651	RNase_Z	ribonuclease Z
TIGR03626	L3_arch	ribosomal protein uL3
TIGR03627	uS9_arch	ribosomal protein uS9
TIGR03628	arch_S11P	ribosomal protein uS11
TIGR03629	uS13_arch	ribosomal protein uS13
TIGR03636	uL23_arch	ribosomal protein uL23
TIGR03653	uL6_arch	ribosomal protein uL6
TIGR03665	arCOG04150	arCOG04150 universal archaeal KH domain protein
TIGR03670	rpoB_arch	DNA-directed RNA polymerase subunit B
TIGR03671	cca_archaeal	CCA-adding enzyme
TIGR03672	rpl4p_arch	50S ribosomal protein uL4
TIGR03673	uL14_arch	50S ribosomal protein uL14
TIGR03674	fen_arch	flap structure-specific endonuclease
TIGR03677	eL8_ribo	ribosomal protein eL8
TIGR03680	eif2g_arch	translation initiation factor 2, gamma subunit
TIGR03683	A-tRNA_syn_arch	alanine--tRNA ligase
TIGR03684	arCOG00985	arCOG04150 universal archaeal PUA-domain protein
TIGR03722	arch_KAE1	universal archaeal protein Kae1

Legends for Suppl. Tables 1-3, Tables 5-7, Table 9, Table 12

Please note that these Suppl. Tables are provided in separate excel files.

Supplementary Table 1. Statistics of high-quality (completeness of > 90% and contamination of < 5%) and medium-quality (completeness of > 70% and contamination of < 10%) archaeal bins recovered from 13 mangrove sediment samples.

Supplementary Table 2. BLAST results generated by searching reads from TDP7 and TDP9 against H03B1/HK01M/HK01B/HK02M1/HK02M2 McrA sequence using the BLASTX method of DIAMOND (cutoffs: e-value <1e-5, >85% identity, alignment length >80%).

Supplementary Table 3. Environmental distribution of the '*Ca.* Methylarchaeales'. The number and percent coverage of reads mapping to the '*Ca.* Methylarchaeales' 16S rRNA genes (cutoffs: ≥ 97% identity and ≥50% alignment length) are shown. The number and percent coverage of reads mapping to the '*Ca.* Methylarchaeales' *mcrA* genes (cutoffs: ≥ 85% nucleotide identity and ≥50% alignment length) are presented. The ID, sampling habitat, location, and size of the dataset are provided. ND, not detected.

Supplementary Table 5. The 16S rRNA gene similarity between the '*Ca.* Methylarchaeales' and *Nitrososphaeria* that was obtained by pairwise comparison of the nucleotide sequences.

Supplementary Table 6. The average amino acid identity (AAI) between the '*Ca.* Methylarchaeales' and *Nitrososphaeria* genomes that was obtained by pairwise comparison of orthologous genes.

Supplementary Table 7. The genes used for metabolic reconstruction in this study. The corresponding genes are also presented in the previously reported JZ-2-bin_220 for comparison.

Supplementary Table 9. Reference archaeal genomes used in this study and distribution of genes for Mcr complex and energy-conserving complexes in archaea.

Supplementary Table 12. Methyl-coenzyme M reductase genes (*mcrABCDG*) identified by searching against Pfam using HMMER.

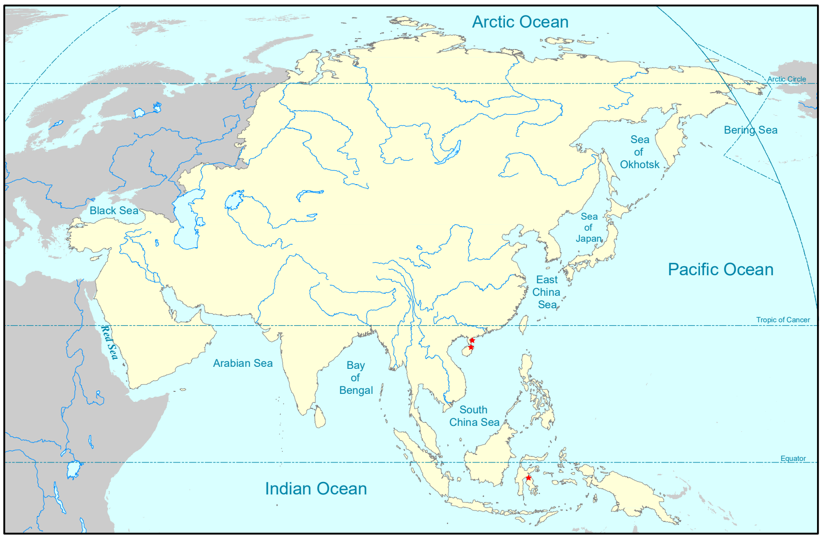

Techeng Island, Guangdong, China
Lon: 110°26′19.67″E
Lat: 21°9′10.80″N

Dongzhai Harbour, Haikou, China
Lon: 110°34′33.6″E
Lat: 19°56′57.0″N

Lake Towuti in Indonesia: South Sulawesi
Lon: 121°29'37.7"E
Lat: 2°46'21.4"S

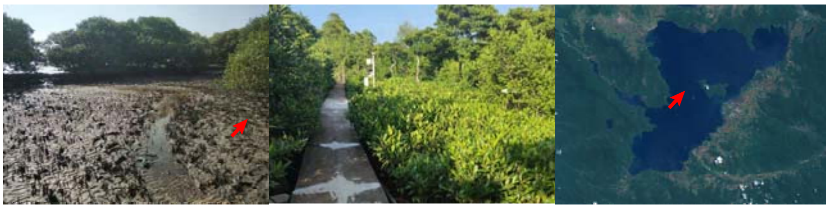

Sample sites

Site name	Interval (m)	Size (GB)	Platform
Techeng Island, Guangdong, China			
H02S	0.15-0.20	60	HiSeq 2500
H02M	0.40-0.45	60	HiSeq 2500
H02B	0.95-1.00	60	HiSeq 2500
H03S	0.15-0.20	60	HiSeq 2500
H03M	0.40-0.45	60	HiSeq 2500
H03B	0.95-1.00	60	HiSeq 2500
Dongzhai Harbour, Haikou, China			
HK01S	0.15-0.20	100	HiSeq 2500
HK01M	0.40-0.45	100	HiSeq 2500
HK01B	0.95-1.00	100	HiSeq 2500
HK02S	0.15-0.20	100	HiSeq 2500
HK02M	0.40-0.45	100	HiSeq 2500
HK03S	0.15-0.20	100	HiSeq 2500
HK03M	0.40-0.45	100	HiSeq 2500
Lake Towuti in Indonesia: South Sulawesi			
TDP7	NULL	41	NovaSeq 6000
TDP9	NULL	37	NovaSeq 6000

Supplementary Fig. 1. Locations of the sampling stations for metagenomes.

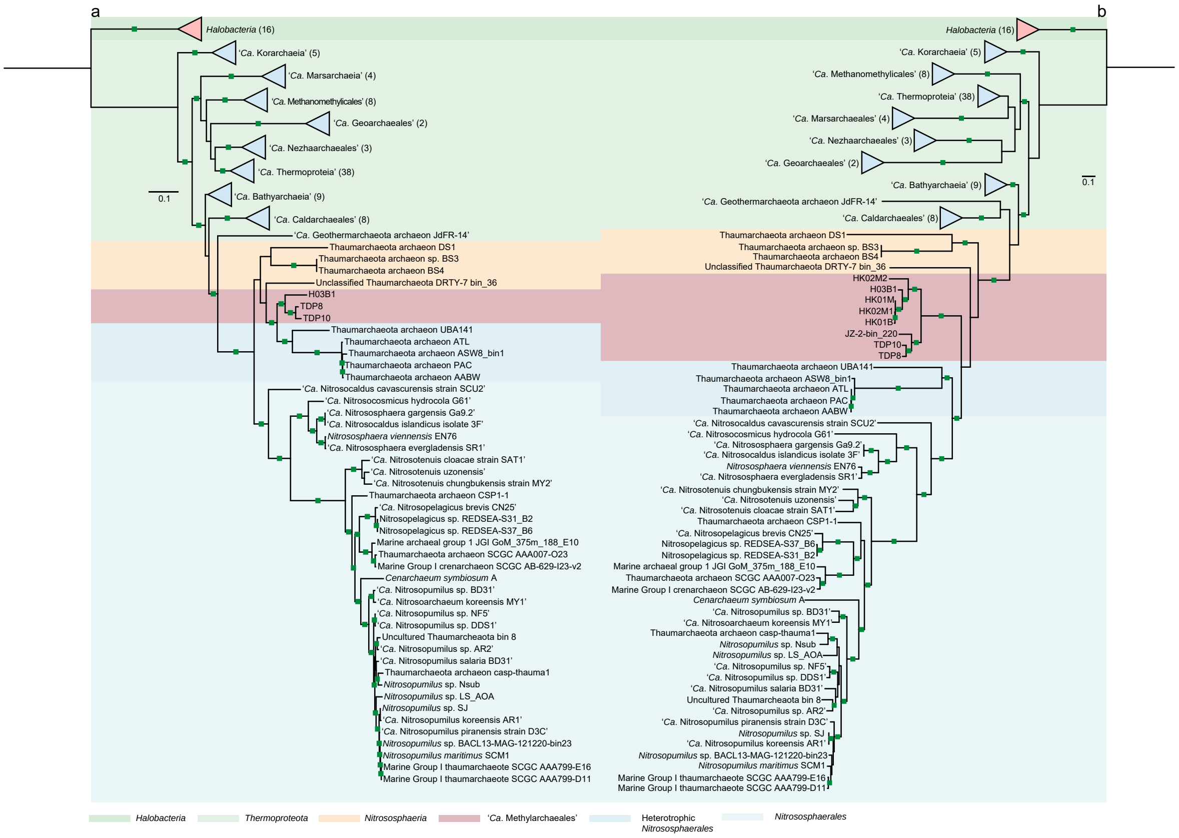

Supplementary Fig. 2. Phylogenetic placement of the '*Ca*. Methylarchaeales'. **a.** Maximum-likelihood tree of concatenated 16S and 23S rRNA genes inferred using IQ-TREE [-m TEST (GTR+F+I+G4), -bb 1000]. The *Halobacteria* class was used as an outgroup. The 23S rRNA gene is missing in TDP8 genome. **b.** Maximum-likelihood tree of a concatenated set of 122 archaeal-specific marker genes inferred with IQ-TREE (LG+F+I+G4, -bb 1000), showing the placement of the '*Ca*. Methylarchaeales' in *Thermoproteota* phylum. The *Halobacteria* class was used as an outgroup. The bootstrap support values ≥ 95 are indicated with green filled squares.

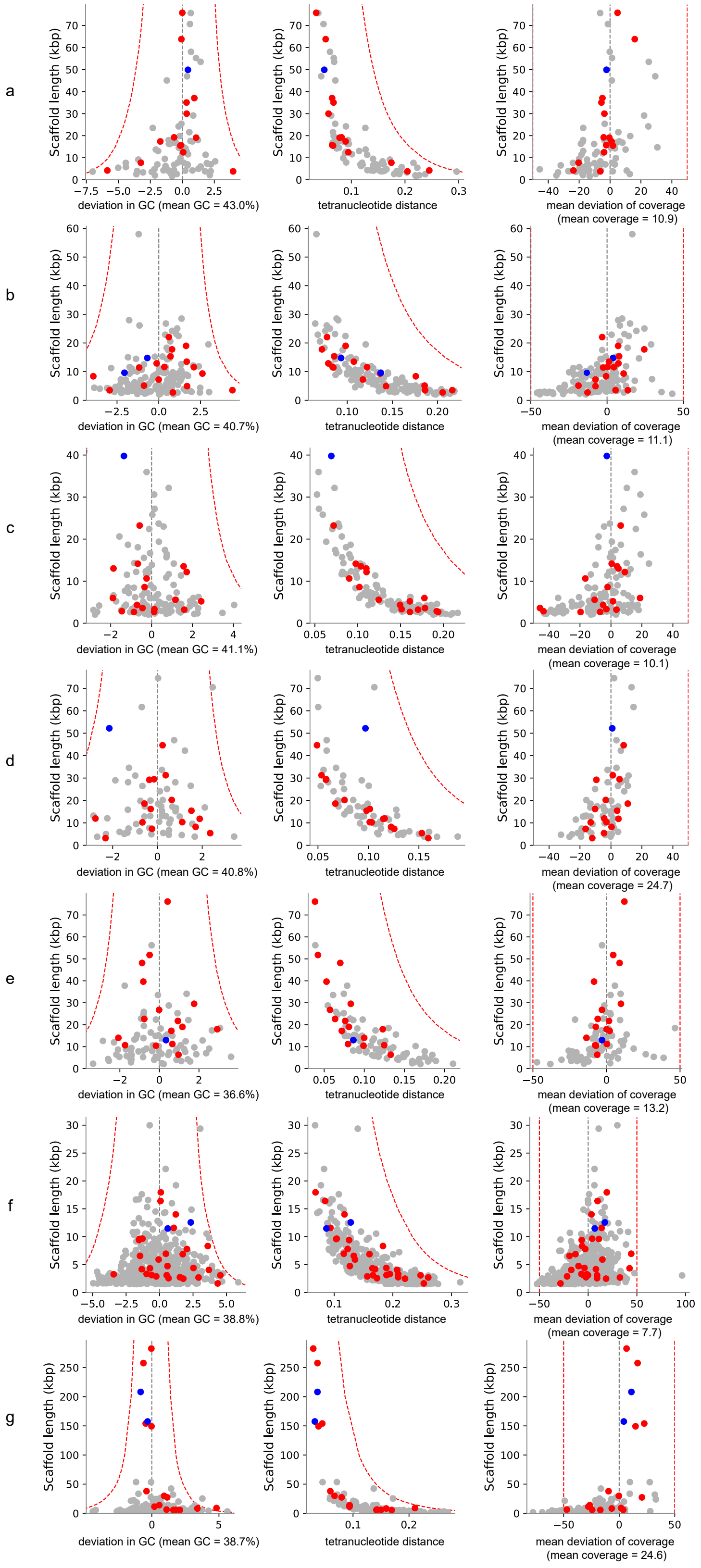

Supplementary Fig. 3. Statistical characteristics of contigs constituting the '*Ca*. Methylarchaeales' genomes (a, H03B1; b, HK01M; c, HK01B; d, HK02M1; e, HK02M2; f, TDP8; g, TDP10.). The contig length was plotted as a function of the GC deviation, tetranucleotide distance and coverage deviation. Each dot in these scatterplots represents a contig. Contigs containing the *mcrABG* and *hdrDE* genes were labeled with blue; contigs containing the *vhtAGC* genes were labeled with purple; red dots are contigs with other methane metabolism-related genes. The dashed red lines indicate the 95th percentile of a typical genome computed with RefineM. *mcr*, methyl-coenzyme M reductase; *hdr*, heterodisulfide reductase; *vht*, methanophenazine-reducing hydrogenase.

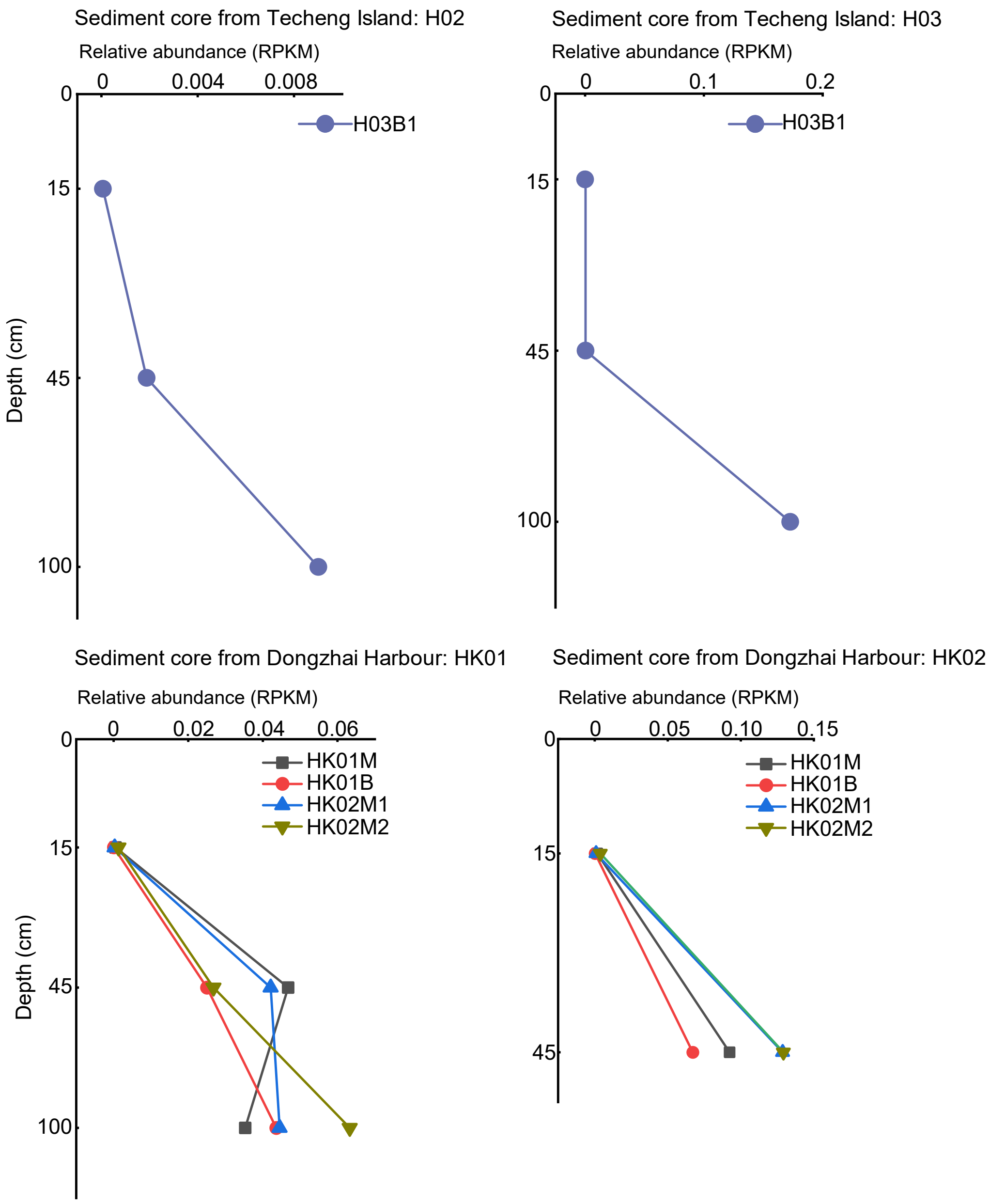

Supplementary Fig. 4. Relative abundance of the '*Ca*. Methylarchaeales' genomes in sediment cores collected from their sampling sites (mangrove wetlands in Techeng Island and Dongzhai Harbour). Relative abundance was represented by reads per kbp of each genome per Mbp of each metagenome (RPKM; (reads recruited to a genome/(length of genome in bp/1,000))/(total bp in metagenome/1,000,000)).

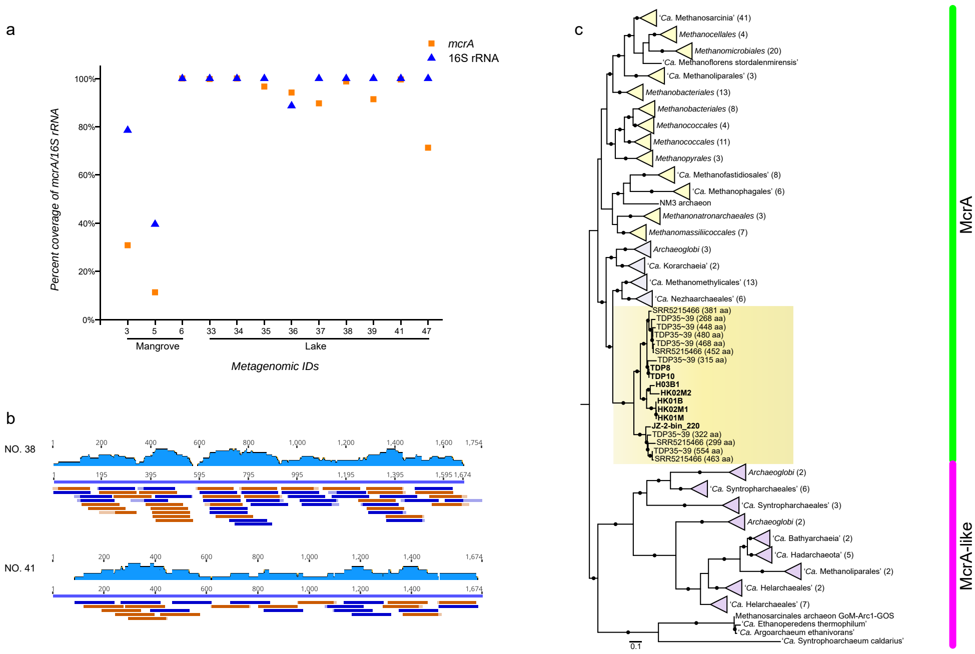

Supplementary Fig. 5. Comparison of the '*Ca*. Methylarchaeales' 16S rRNA and *mcrA* genes to sequence reads from metagenomes generated from mangrove and lake sediment. **a.** The percent coverage information of reads recruited to '*Ca*. Methylarchaeales' 16S rRNA and *mcrA* genes in 13 metagenomes. The highest percent coverage in each metagenome was presented. The metagenomic IDs on X axis correspond to those in Supplementary Table 3. **b.** Metagenomic reads mapping to the *mcrA* gene of the '*Ca*. Methylarchaeales'. Samples NO. 38 and NO. 41 represent metagenomes generated from Lake Towuti and Gulf of Boni, respectively. Detailed information about metagenomes is provided in Supplementary Table 3. Reads in blue and dark orange are reads mapped in forward and reverse orientation, respectively. **c.** Phylogenetic tree of the methyl-coenzyme M reductase (Mcr)/Mcr-like subunit A constructed using IQ-TREE (-m TEST LG+F+I+G4, -bb 1000), showing the position of McrA fragments (> 250 aa) recovered from metagenomes (in yellow). These McrA fragments clustered with McrA sequences of the '*Ca*. Methylarchaeales' MAGs (in bold). SRR5215466, sediment metagenome from Gulf of Boni, Indonesia; TDP, ID 35-39 metagenomes generated from sediments of Lake Towuti, Indonesia (Supplementary Table 3).

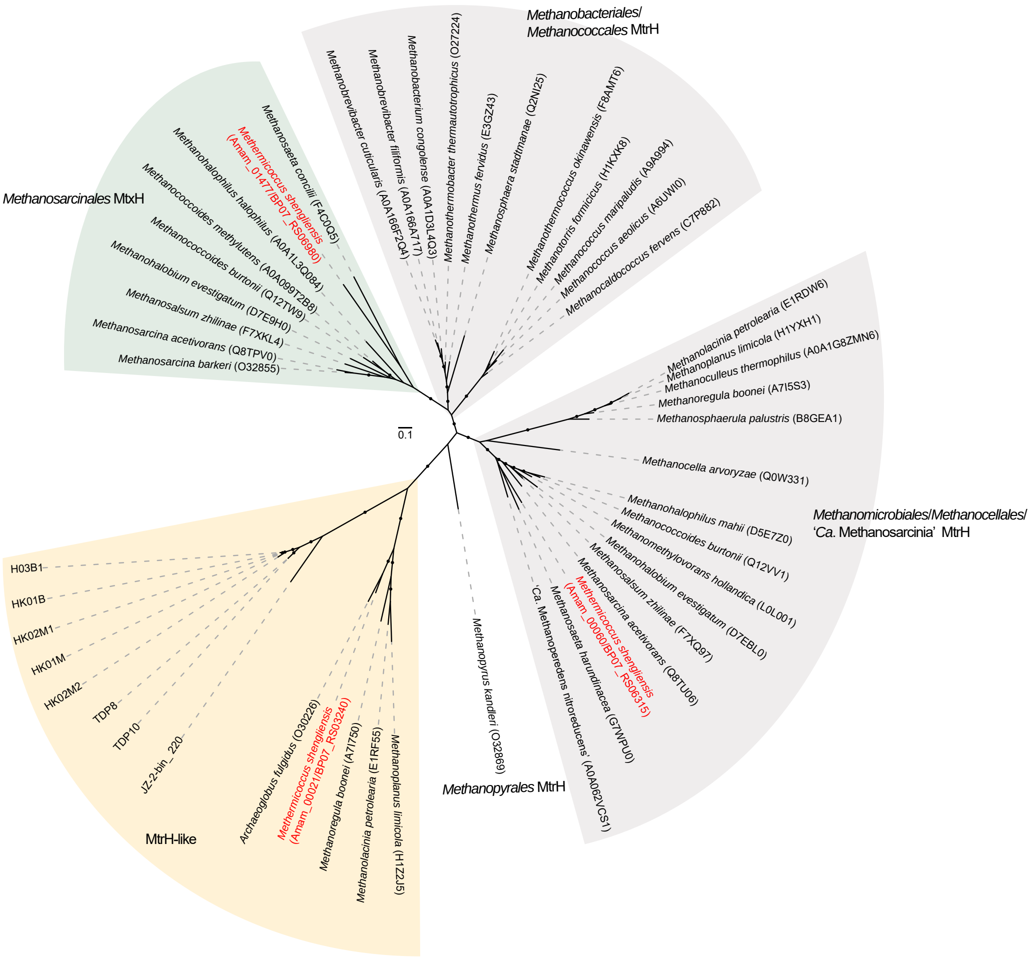

Supplementary Fig. 6. Phylogenetic analysis of methyl-tetrahydromethanopterin:coenzyme M methyltransferase subunit H. MtrH reference sequences were derived from a previous study [13]. Ultrafast bootstraps values ≥ 95 are indicated with black dots.

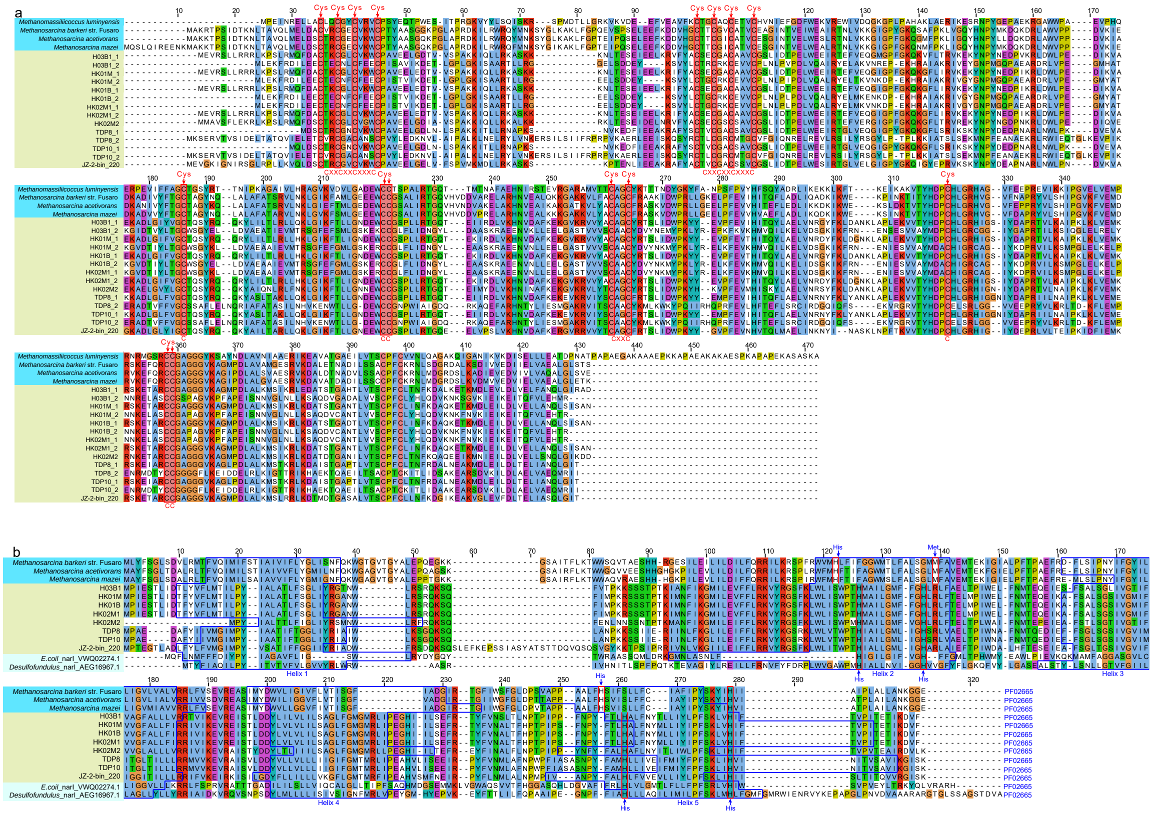

Supplementary Fig. 7. Multiple sequence alignment of heterodisulfide reductase subunit D (HdrD) and E (HdrE) sequences. **a.** Comparison of the '*Ca*. Methylarchaeales' HdrD with known *Methanosarcina* and *Methanomassiliicoccus* HdrD. Cysteines which interact with Fe-S clusters and are located in cysteines-rich motifs are labeled with red boxes [14]. **b.** Comparison of the '*Ca*. Methylarchaeales' HdrE with *Methanosarcina* HdrE and bacterial NarI. The blue box represents the transmembrane region. Five transmembrane helices are indicated by blue boxes and arrows denote locations of histidine residues which can bind with heme groups [15].

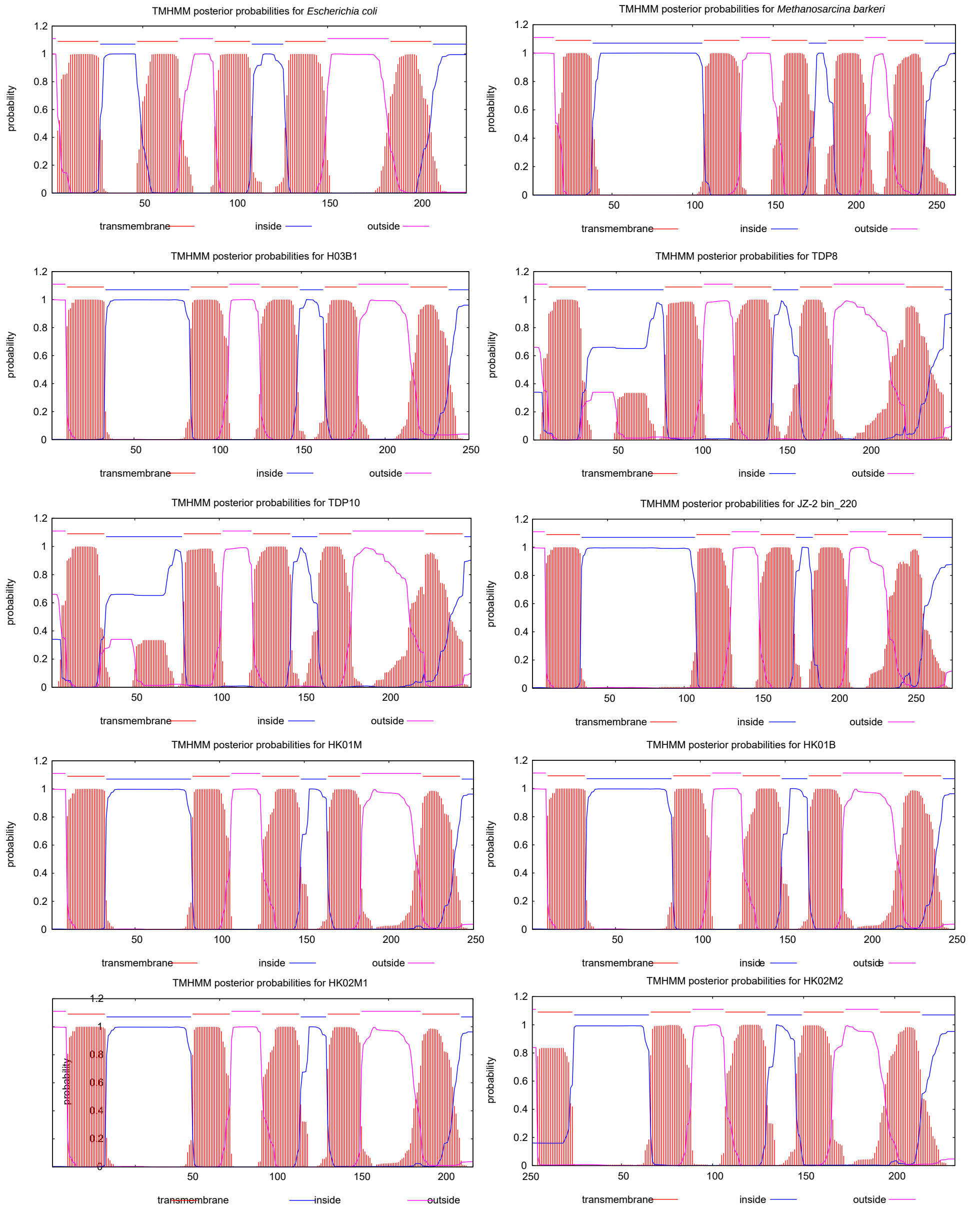

Supplementary Fig. 8. Prediction of transmembrane helices in the ‘*Ca*. Methylarchaeales’ heterodisulfide reductase subunit E (HdrE) sequences using TMHMM Server v. 2.0 (http://www.cbs.dtu.dk/services/TMHMM/). The *b*-type cytochrome of *Escherichia coli* (VWQ02274.1) and *Methanosarcina barkeri* HdrE (P96796.2) were used as controls.

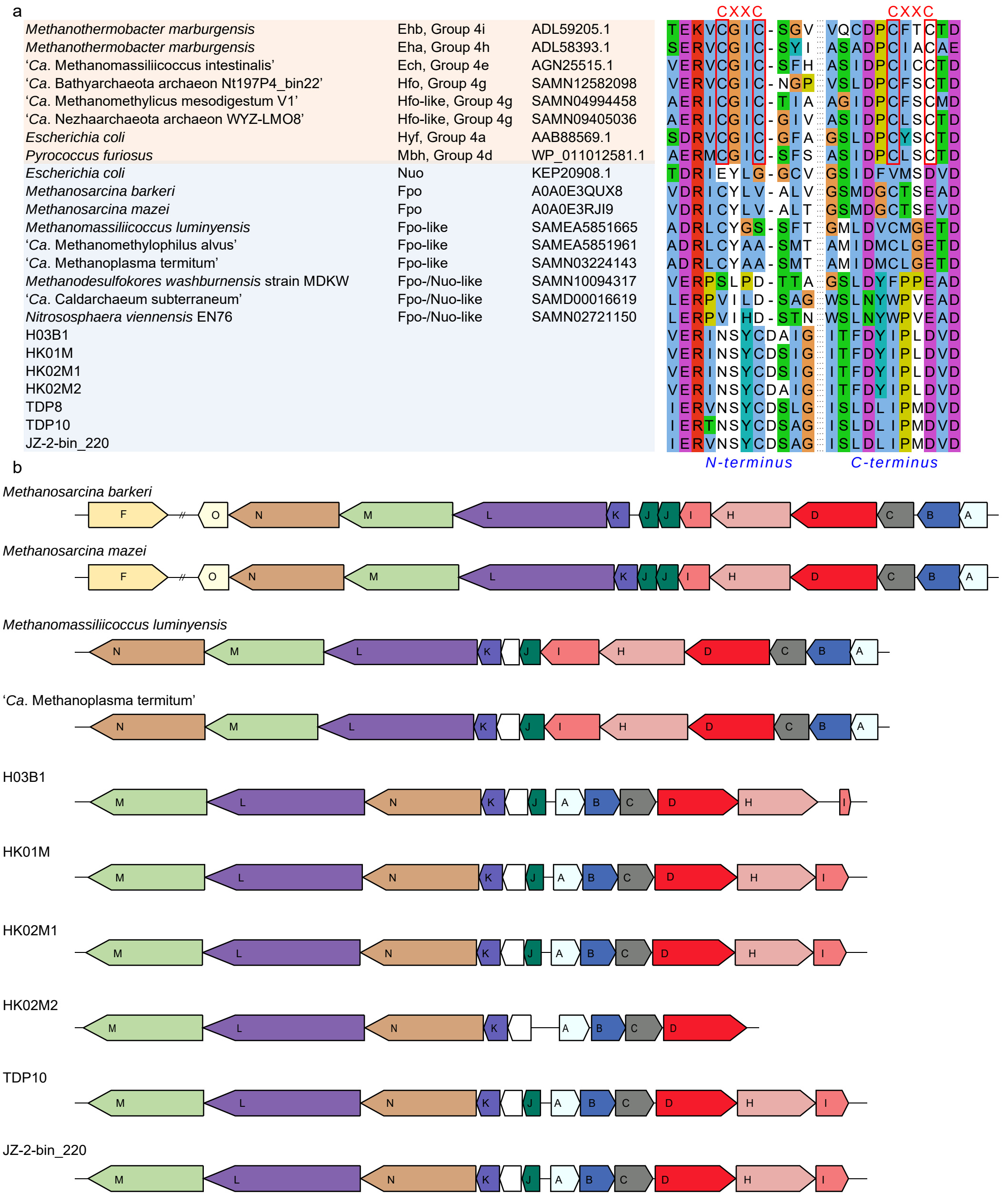

Supplementary Fig. 9. Comparison of the '*Ca*. Methylarchaeales' $F_{420}H_2$ dehydrogenase-like (Fpo-like) with known Fpo, Fpo-like and group 4 [NiFe] hydrogenase. **a.** Sequence alignment of the large subunits of Fpo, Fpo-like and group 4 [NiFe] hydrogenase. The [NiFe]-binding motifs of group 4 [NiFe] hydrogenase are indicated by red boxes. **b.** Organization of the gene clusters encoding Fpo/Fpo-like complexes.

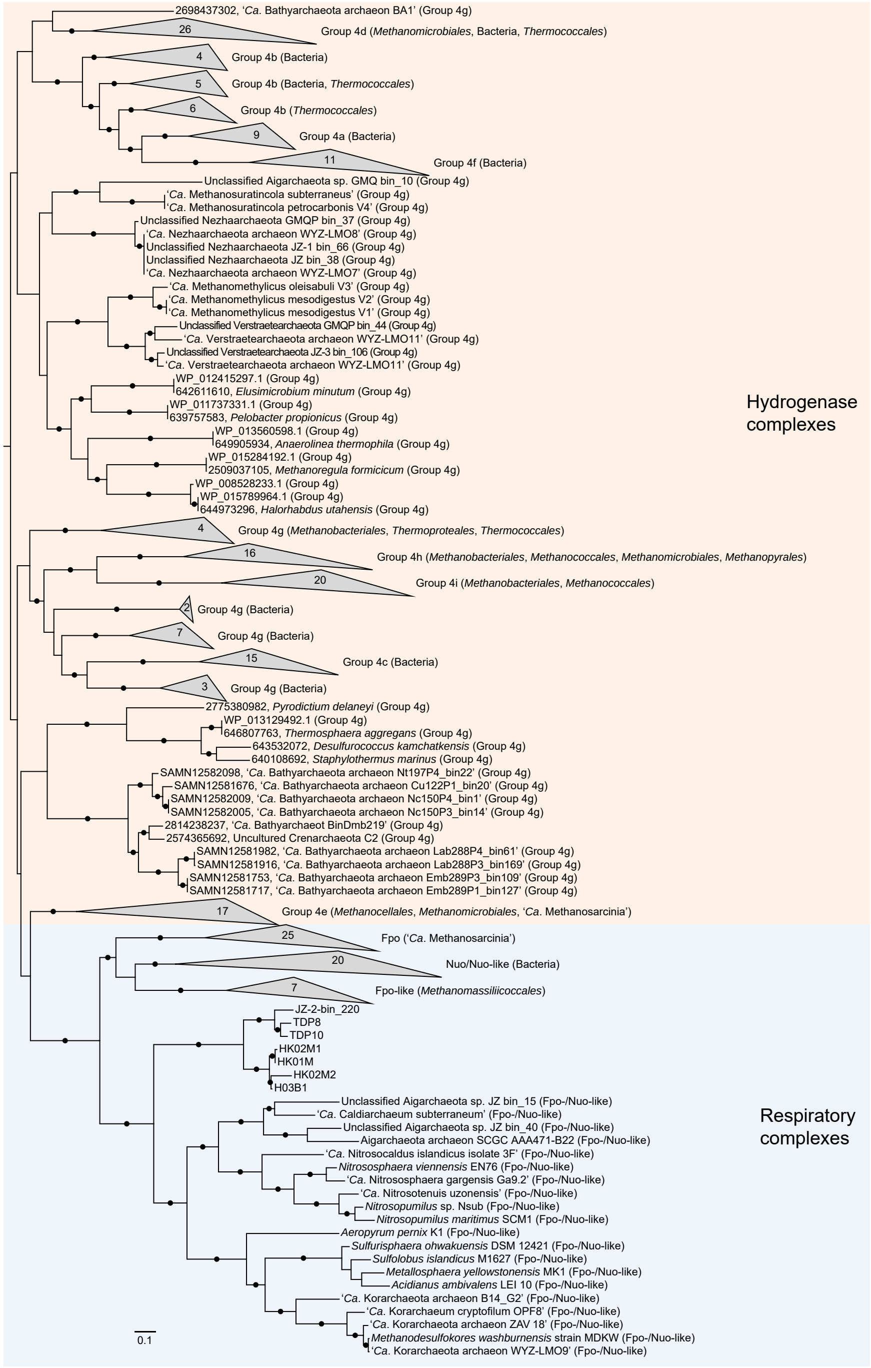

Supplementary Fig. 10. Phylogeny of the catalytic subunit of selected group 4 [NiFe] hydrogenases and their homologs in the respiratory complexes (Nuo, Fpo and Fpo-like). The catalytic subunit of selected group 4 [NiFe] hydrogenases sequences were downloaded from HydDB [16]. Ultrafast bootstraps values ≥ 95 are indicated with black dots.

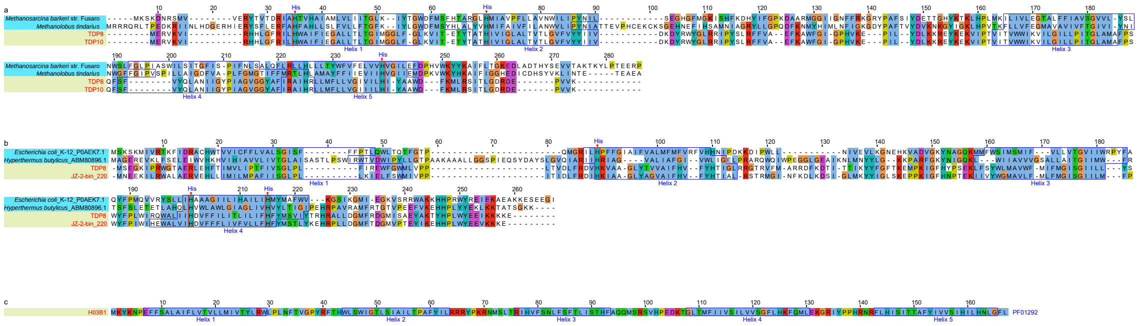

Supplementary Fig. 11. Multiple sequence alignment of methanophenazine-reducing hydrogenase cytochrome *b* subunit (VhtC) sequences (a) and formate dehydrogenase gamma subunit (FdhC) sequences (b, c). **a.** Comparison of the '*Ca*. Methylarchaeales' VhtC with that of cultured *Methanosarcina barkeri* str. Fusaro and *Methanolobus tindarius*. **b.** Comparison of the '*Ca*. Methylarchaeales' FdhC with that of *E.coil* k-12 and *Hyperthermus butylicus*. **c.** The putative FdhC sequence from the H03B1 genome identified using HMMER based on prokaryotic cytochrome b_{561} domain (PF01292). It possesses five transmembrane helix regions which is different from *E.coil* k-12 FdhC. The transmembrane helix regions are indicated by blue boxes. The histidine residues that bind heme are labeled with red boxes.

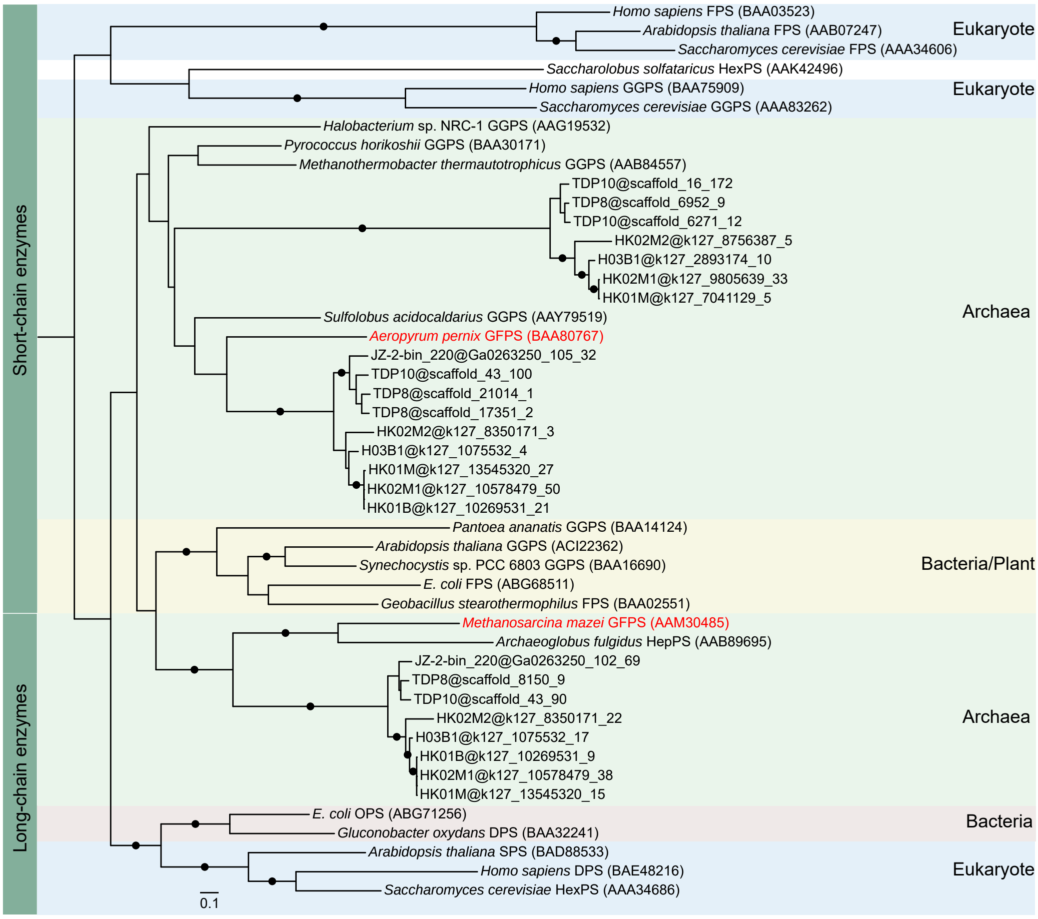

Supplementary Fig. 12. Phylogenetic tree of (all-E) prenyl diphosphate synthases. The reference sequences were derived from a previous study [17]. The geranylfarnesyl diphosphate synthase (GFPS) which is involved in methanophenazine biosynthesis is indicated with red. Gene numbers indicate protein accession IDs from NCBI. Ultrafast bootstraps values ≥ 95 are indicated with black dots.

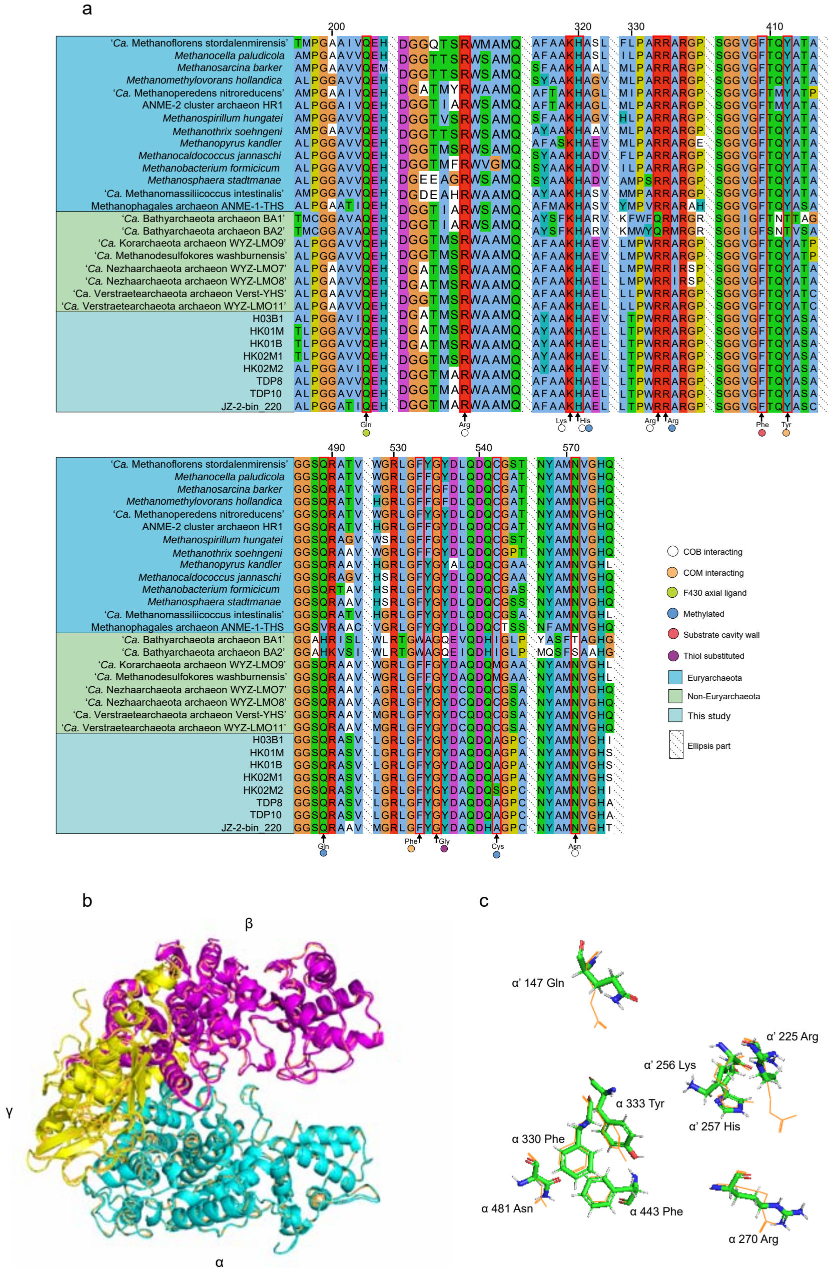

Supplementary Fig. 13. Conserved active sites of McrA (a) and structural model of the H03B1 Mcr complex (b and c). **a.** Key amino acid residues identified by Ermler et al. [11] including binding sites for CoM, CoB, and F_{430} are shown. **b.** Models of the α (cyan), β (magenta), and γ (yellow) subunits of H03B1 Mcr overlaid on the corresponding subunits (orange) of the *Methanosarcina barkeri* crystal structure (PDB ID: 1e6y). **c.** Model of the activate sites of the McrA subunits (sticks) of H03B1 superimposed onto McrA (orange ribbon) of *Methanosarcina barkeri*.

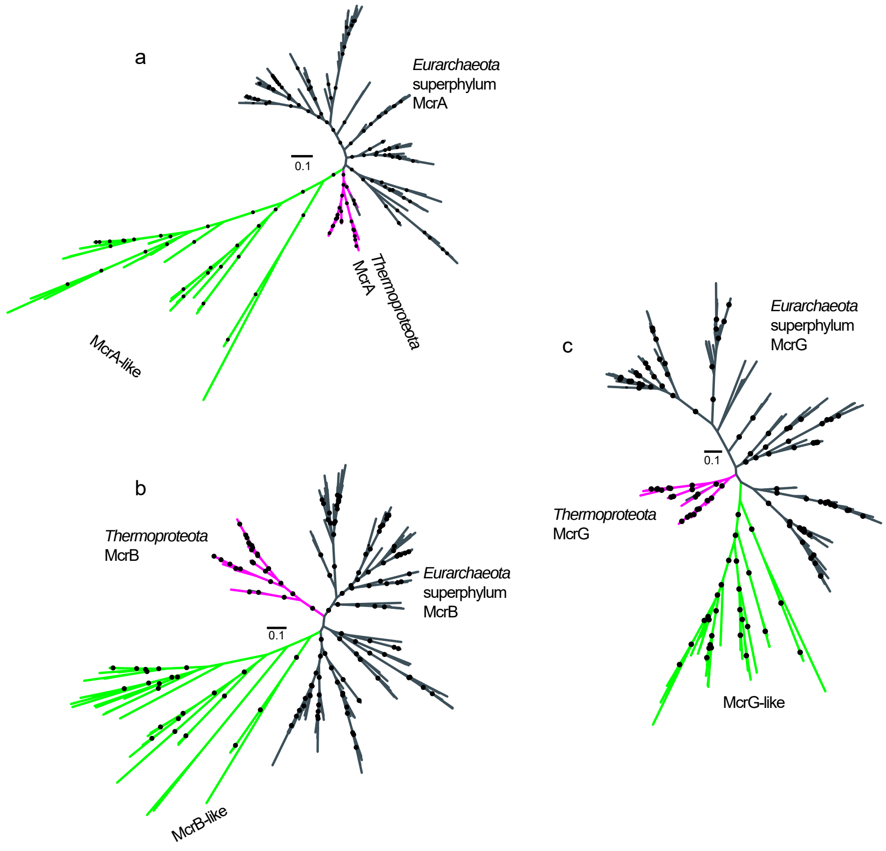

Supplementary Fig. 14. Phylogenetic trees of the methyl-coenzyme M reductase (Mcr)/ Mcr-like complex subunits (a McrA/McrA-like, b McrB/McrB-like, and c McrG/McrG-like) constructed using IQ-TREE. The trees include 167 archaeal genomes with Mcr/Mcr-like complex. The model used was LG+F+I+G4 for *mcrA* and *mcrB* genes, and LG+G4 for *mcrG* gene. Members of *Euryarchaeota* superphylum and *Thermoproteota* phylum are represented by dark-green branches and pink branches, respectively. Mcr-like complex is indicated with green branches. Ultrafast bootstraps values ≥ 95 are indicated with squares.

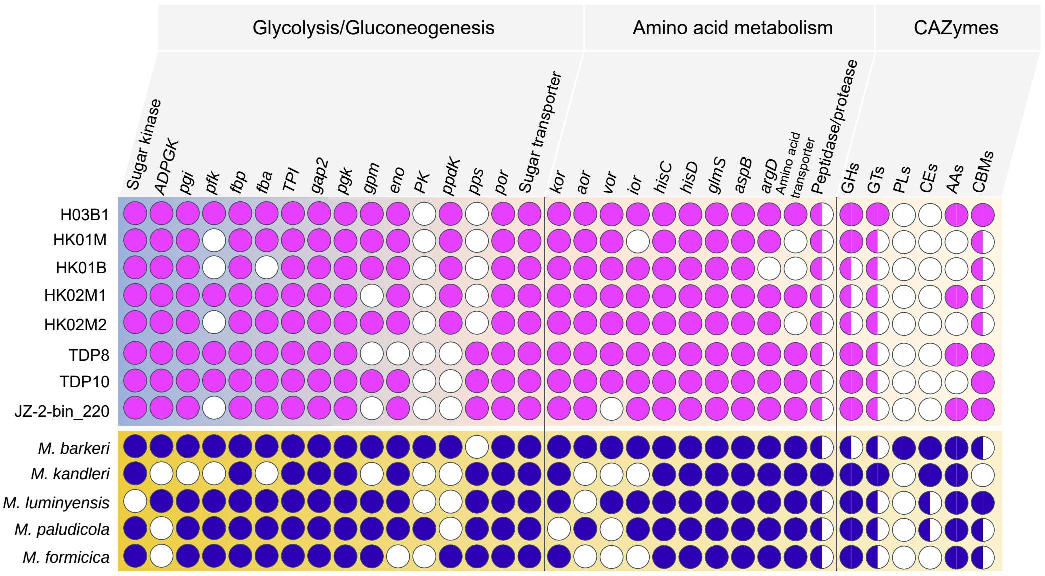

Supplementary Fig. 15. Distribution of key genes involved in glycolysis/gluconeogenesis, and amino acid and carbohydrate metabolism in the '*Ca*. Methylarchaeales' and cultivated methanogens. The semicircle indicates the kind of enzymes that are predicted to be either extracellular or intracellular. The filled circle indicates the enzymes that are predicted to be intracellular. The empty circle represents the absence of these enzymes. For abbreviation of the genes in glycolysis/gluconeogenesis and amino acid metabolism, their full gene name is presented in Supplementary Table 7. CAZymes, carbohydrate-active enzymes; *PK*, pyruvate kinase; GHs, glycoside hydrolases; GTs, glycosyl transferases; PLs, polysaccharide lyases; CEs, carbohydrate esterases; AAs, auxiliary activities; CBMs, carbohydrate-binding modules; *M. barkeri*, *Methanosarcina barkeri* str. Fusaro; *M. kandleri*, *Methanopyrus kandleri*; *M. luminyensis*, *Methanomassiliicoccus luminyensis*; *M. paludicola*, *Methanocella paludicola*; *M. formicica*, *Methanoregula formicica*.

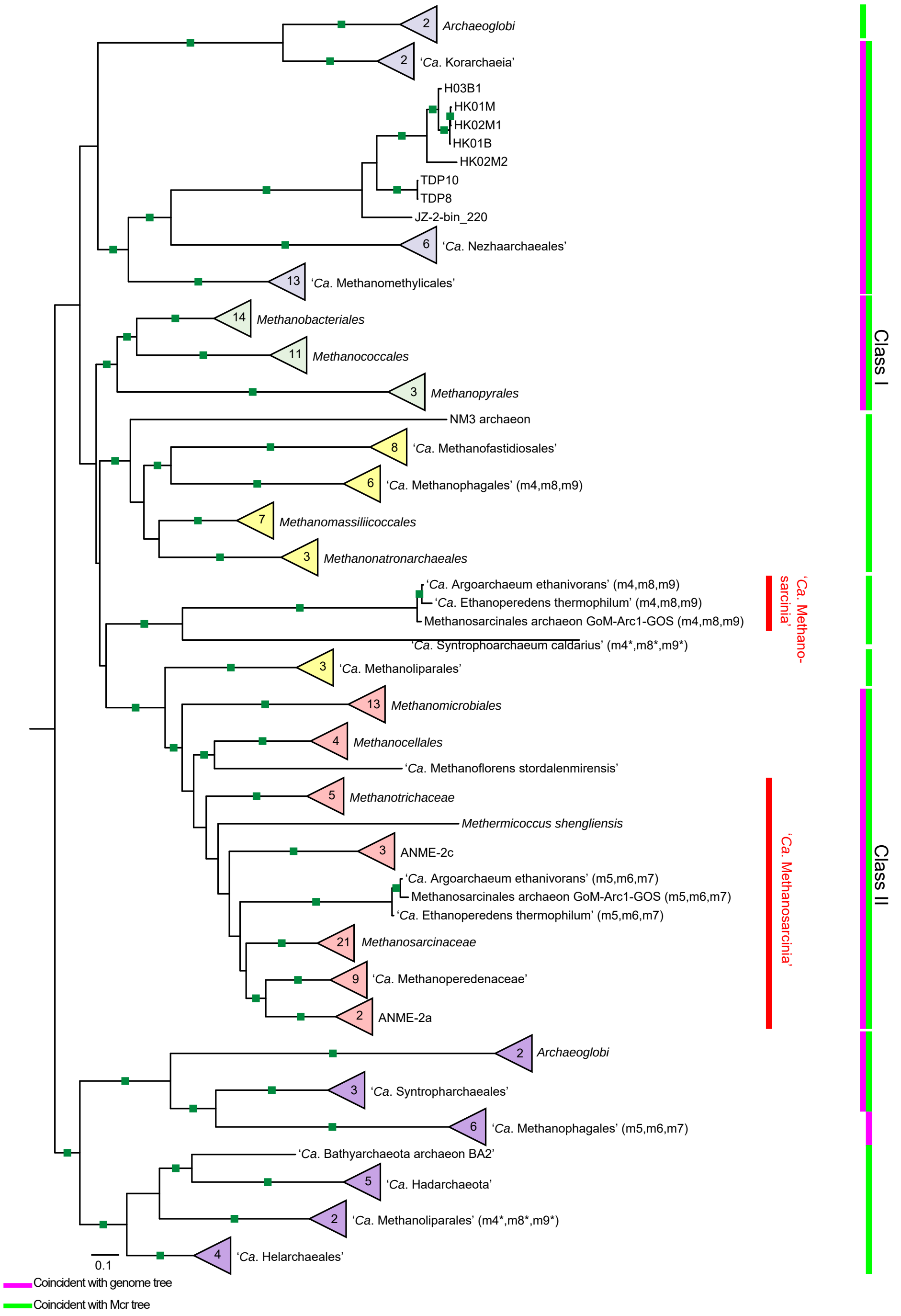

Supplementary Fig. 16. The maximum-likelihood phylogeny (IQ-TREE, LG+C60+F+G) based on a concatenation of markers m4 to m9 from 164 *mcr*-containing archaeal genomes. The bootstrap support values ≥ 95 are indicated with green filled squares. For the '*Ca.* Methanophagales', '*Ca.* Argoarchaeum ethanivorans', Methanosarcinales archaeon GoM-Arc1-GOS and '*Ca.* Ethanoperedens thermophilum', their markers are divided into two incongruent parts which are individually concatenated and displayed in front of the corresponding branches. As for extra copies of m4, m8 and m9 in '*Ca.* Syntrophoarchaeum caldarius' and '*Ca.* Methanoliparales', they are individually concatenated and labeled with asterisk. Vertical lines in front of the phylogeny indicate that the corresponding sections are congruent with the species tree (pink) and/or the concatenated McrABG/McrABG-like tree (green) (Fig 4).

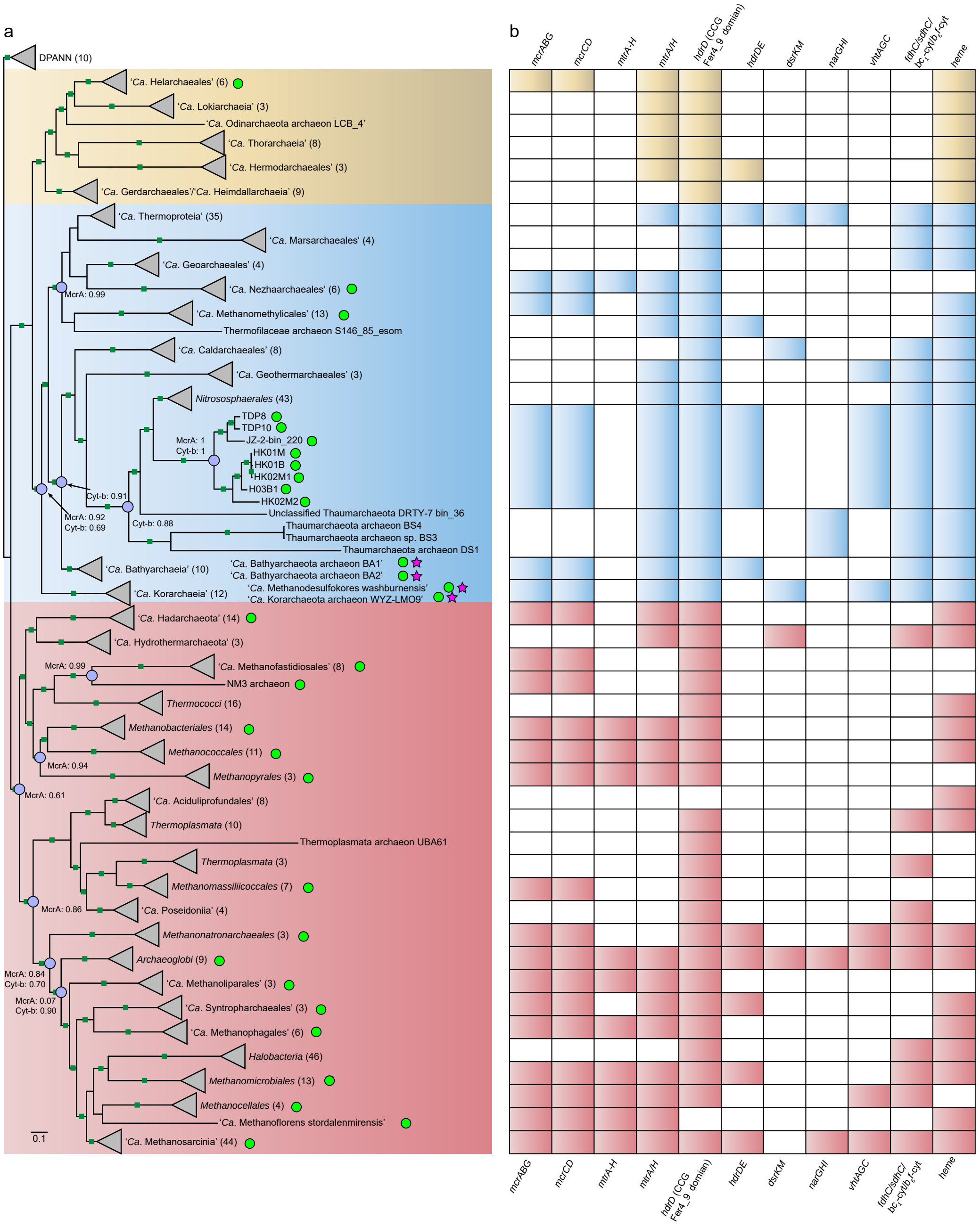

Supplementary Fig. 17. Genome tree and distribution of genes for Mcr complex and energy-conserving complexes in archaea. **a.** Species tree based on concatenated 122 archaeal-specific marker proteins using 426 representative archaeal genomes. The tree is inferred with IQTREE (LG+C60+F+G, -bb 1000) and rooted between DPANN and all other archaea. The bootstrap support values ≥ 95 are indicated with green squares. The branches with the Mcr/Mcr-like complex are marked with green circle. Asterisk in front of BA1, BA2, WYZ LMO9, and MDKW indicates that these genomes lack the HdrDE complex. **b.** Presence and absence of key genes related to cytochrome *b*-containing methanogens in main archaeal lineages. For mtrA-H, it was regarded as present if ≥80% of the subunit genes constituting these complexes were identified. For other complexes, they were regarded as present only when all subunit genes for these complexes were found. As for heme biosynthetic pathway, it was regarded as present if more than two of three key enzymes (Ahb-NirDH, Ahb-NirJ1 and Ahb-NirJ2) were identified in a genome. For archaeal lineages (order or phylum), if there is at least one genome in a lineage that was found to contain the genes above, the corresponding grid is shaded with color. Presence probability of McrA or *b*-type cytochrome in some nodes computed by ALE are indicated.

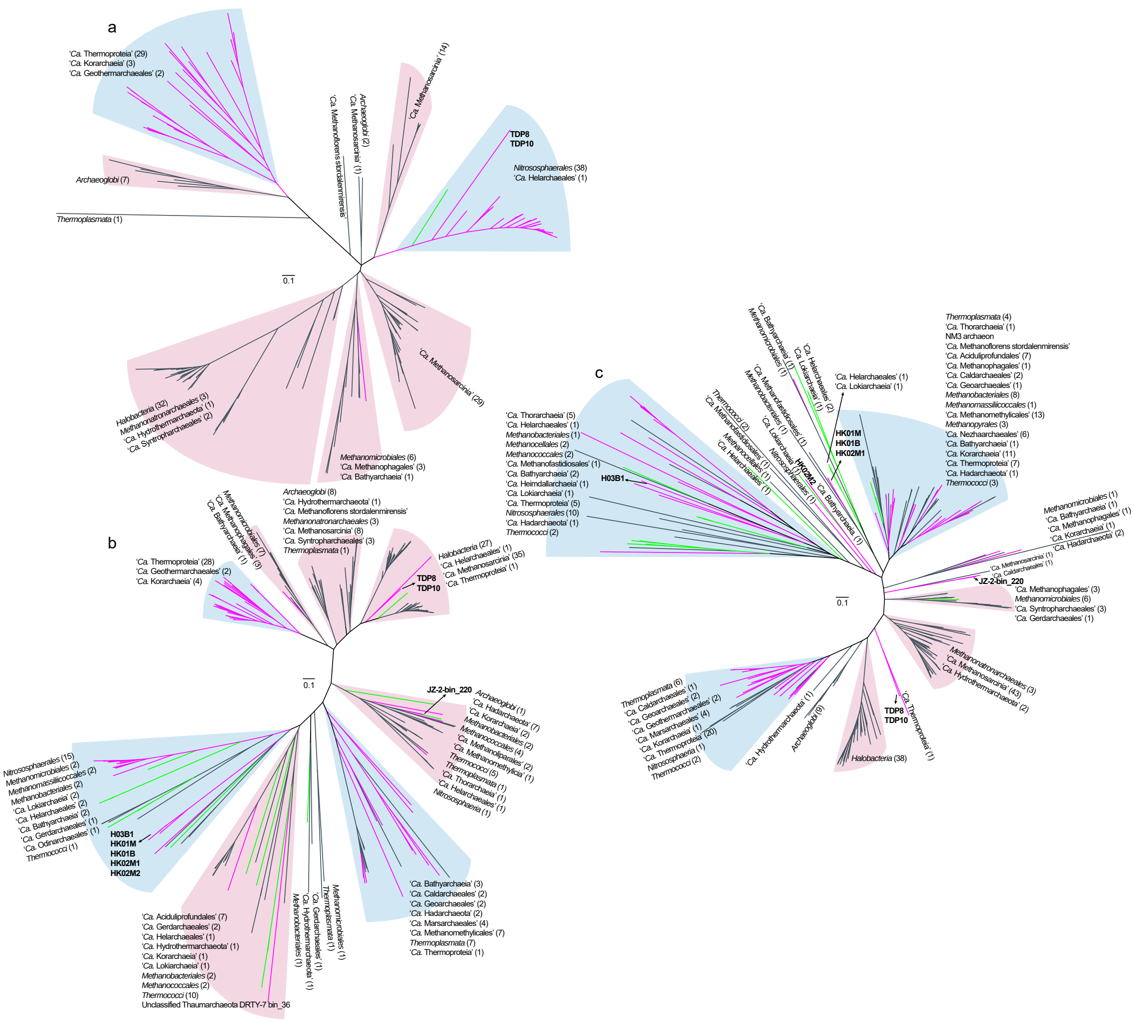

Supplementary Fig. 18. Phylogenetic analyses of Ahb-NirDH (a), Ahb-NirJ1 (b), and Ahb-NirJ2 (c) involved in heme biosynthetic pathway. The Unrooted maximum-likelihood trees were inferred with IQ-TREE (-m TEST, -bb 1000). The dark-green branches represent members of *Euryarchaeota* superphylum while the pink branches represent members of *Thermoproteota* phylum, and the green branches represent members of *Asgardarchaeota*. Figures in bracket indicate number of sequences.

Reference

1. Zhang Y. I-TASSER server for protein 3D structure prediction. BMC Bioinf. 2008;9:40.
2. Langmead B, Salzberg SL. Fast gapped-read alignment with Bowtie 2. Nat Methods. 2012;9:357.
3. Li H, Handsaker B, Wysoker A, Fennell T, Ruan J, Homer N, et al. The sequence alignment/map format and samtools. Bioinformatics. 2009;25:2078-2079.
4. Agusto LE, Fratini S, Jimenez PJ, Quadros A, Cannicci S. Structural characteristics of crab burrows in Hong Kong mangrove forests and their role in ecosystem engineering. Estuarine, Coastal Shelf Sci. 2021;248:106973.
5. Gao Y, Zhou J, Wang L, Guo J, Feng J, Wu H, et al. Distribution patterns and controlling factors for the soil organic carbon in four mangrove forests of China. Glob Ecol Conserv. 2019;17:e00575.
6. Quast C, Pruesse E, Yilmaz P, Gerken J, Schweer T, Yarza P, et al. The SILVA ribosomal RNA gene database project: improved data processing and web-based tools. Nucleic Acids Res. 2012;41:D590-D596.
7. Li H. Aligning sequence reads, clone sequences and assembly contigs with BWA-MEM. arXiv. 2013.
8. Yarza P, Yilmaz P, Pruesse E, Glöckner FO, Ludwig W, Schleifer KH, et al. Uniting the classification of cultured and uncultured bacteria and archaea using 16S rRNA gene sequences. Nat Rev Microbiol. 2014;12:635-645.
9. Storbeck S, Rolfes S, Raux Deery E, Warren MJ, Jahn D, Layer G. A novel pathway for the biosynthesis of heme in archaea: genome-based bioinformatic predictions and experimental evidence. Archaea. 2010;2010.
10. Kühner M, Haufschildt K, Neumann A, Storbeck S, Streif J, Layer G. The alternative route to heme in the methanogenic archaeon *Methanosarcina barkeri*. Archaea. 2014;2014.
11. Ermler U, Grabarse W, Shima S, Goubeaud M, Thauer RK. Crystal structure of methyl-coenzyme M reductase: the key enzyme of biological methane formation.

Science. 1997;278:1457-1462.

12. Chaumeil PA, Mussig AJ, Hugenholtz P, Parks DH. GTDB-Tk: a toolkit to classify genomes with the genome taxonomy database. Bioinformatics. 2019;36:1925-1927.
13. Kurth JM, Nobu MK, Tamaki H, de Jonge N, Berger S, Jetten MS, et al. Methanogenic archaea use a bacteria-like methyltransferase system to demethoxylate aromatic compounds. ISME J. 2021;15:3549-3565.
14. Kröninger L, Berger S, Welte C, Deppenmeier U. Evidence for the involvement of two heterodisulfide reductases in the energy-conserving system of *Methanomassiliicoccus luminyensis*. FEBS J. 2016;283:472-483.
15. Künkel A, Vaupel M, Heim S, Thauer RK, Hedderich R. Heterodisulfide reductase from methanol-grown cells of *Methanosarcina barkeri* is not a flavoenzyme. Eur J Biochem. 1997;244:226-234.
16. Søndergaard D, Pedersen CN, Greening C. HydDB: a web tool for hydrogenase classification and analysis. Sci Rep. 2016;6:1-8.
17. Ogawa T, Yoshimura T, Hemmi H. Geranylfarnesyl diphosphate synthase from *Methanosarcina mazei*: different role, different evolution. Biochem Biophys Res Commun. 2010;393:16-20.
